# Tuberculosis detection and the challenges of integrated care in rural China: A cross-sectional standardized patient study

**DOI:** 10.1371/journal.pmed.1002405

**Published:** 2017-10-17

**Authors:** Sean Sylvia, Hao Xue, Chengchao Zhou, Yaojiang Shi, Hongmei Yi, Huan Zhou, Scott Rozelle, Madhukar Pai, Jishnu Das

**Affiliations:** 1 Department of Health Policy and Management, Gillings School of Global Public Health, University of North Carolina at Chapel Hill, Chapel Hill, North Carolina, United States of America; 2 Center for Experimental Economics in Education, Shaanxi Normal University, Xi’an, China; 3 Institute of Social Medicine and Health Administration, School of Public Health, Shandong University, Jinan, China; 4 School of Advanced Agricultural Sciences, Peking University, Beijing, China; 5 Department of Health and Social Behavior, West China School of Public Health, Sichuan University, Chengdu, China; 6 Freeman Spogli Institute for International Studies, Stanford University, Stanford, California, United States of America; 7 McGill International TB Centre & Department of Epidemiology, Biostatistics and Occupational Health, McGill University, Montreal, Canada; 8 Development Research Group, The World Bank, Washington, D.C., United States of America; Universidad Peruana Cayetano Heredia, PERU

## Abstract

**Background:**

Despite recent reductions in prevalence, China still faces a substantial tuberculosis (TB) burden, with future progress dependent on the ability of rural providers to appropriately detect and refer TB patients for further care. This study (a) provides a baseline assessment of the ability of rural providers to correctly manage presumptive TB cases; (b) measures the gap between provider knowledge and practice and; (c) evaluates how ongoing reforms of China’s health system—characterized by a movement toward “integrated care” and promotion of initial contact with grassroots providers—will affect the care of TB patients.

**Methods/Findings:**

Unannounced standardized patients (SPs) presenting with classic pulmonary TB symptoms were deployed in 3 provinces of China in July 2015. The SPs successfully completed 274 interactions across all 3 tiers of China’s rural health system, interacting with providers in 46 village clinics, 207 township health centers, and 21 county hospitals. Interactions between providers and standardized patients were assessed against international and national standards of TB care. Using a lenient definition of correct management as at least a referral, chest X-ray or sputum test, 41% (111 of 274) SPs were correctly managed. Although there were no cases of empirical anti-TB treatment, antibiotics unrelated to the treatment of TB were prescribed in 168 of 274 interactions or 61.3% (95% CI: 55%–67%). Correct management proportions significantly higher at county hospitals compared to township health centers (OR 0.06, 95% CI: 0.01–0.25, *p* < 0.001) and village clinics (OR 0.02, 95% CI: 0.0–0.17, *p* < 0.001). Correct management in tests of knowledge administered to the same 274 physicians for the same case was 45 percentage points (95% CI: 37%–53%) higher with 24 percentage points (95% CI: −33% to −15%) fewer antibiotic prescriptions. Relative to the current system, where patients can choose to bypass any level of care, simulations suggest that a system of managed referral with gatekeeping at the level of village clinics would reduce proportions of correct management from 41% to 16%, while gatekeeping at the level of the township hospital would retain correct management close to current levels at 37%. The main limitations of the study are 2-fold. First, we evaluate the management of a one-time new patient presenting with presumptive TB, which may not reflect how providers manage repeat patients or more complicated TB presentations. Second, simulations under alternate policies require behavioral and statistical assumptions that should be addressed in future applications of this method.

**Conclusions:**

There were significant quality deficits among village clinics and township health centers in the management of a classic case of presumptive TB, with higher proportions of correct case management in county hospitals. Poor clinical performance does not arise only from a lack of knowledge, a phenomenon known as the “know-do” gap. Given significant deficits in quality of care, reforms encouraging first contact with lower tiers of the health system can improve efficiency only with concomitant improvements in appropriate management of presumptive TB patients in village clinics and township health centers.

## Abstract 2

Chengchao Zhou and colleagues use standardized patients to evaluate tuberculosis diagnosis and care across the three tiers of China's rural health system.

## Author summary

**Why was this study done?:**

- Despite remarkable progress against the disease in recent decades, China still faces a significant tuberculosis (TB) burden, particularly in poor rural areas where patients have been shown to face significant delays in diagnosis compared to urban areas.
- Existing evidence on the quality of TB care in China is based on surveys of individuals ultimately registered with specialized TB clinics; these may not be representative of the quality of TB care provided in rural China, both due to potential poor recall among patients and the fact that surveyed individuals have all been successfully diagnosed with TB.
- There is little to no direct, objective evidence on how presumptive TB patients are managed in China’s rural health system or on factors related to the quality of care that patients receive.
- Recent reforms of China’s health system—in particular a move toward policies encouraging first contact with lower-tier providers—may have important implications for TB patients, yet no prospective studies have examined these potential effects.

**What did the researchers do and find?:**

- This study uses unannounced standardized patients (SPs) in a random sample of providers to provide the first direct evidence on the ability of providers at each of the 3 tiers of China’s rural health system to correctly manage patients presenting with symptoms of presumptive TB.
- Assessed against lenient international and national standards of TB care, 41% SPs were correctly managed overall with significantly lower rates of correct management in village and township facilities compared to county hospitals.
- In clinical vignettes designed to test knowledge and matching cases presented by SPs, the same village and township clinicians correctly managed cases of presumptive TB more than twice as often.
- Simulations of alternative “managed referral” policies at the health system level show that when patients are free to select the tier at which they first seek care, presumptive TB patients are correctly managed 41% of the time, but this rate would (a) fall substantially if patients need to first seek care in village clinics and (b) would stay approximately the same with managed referrals from the township level.

**What do these findings mean?:**

- There are significant deficits in the management of presumptive TB patients among village clinics and township health centers in rural areas.
- Policy attention should first focus on quality improvement among grassroots providers before moving forward with reforms to encourage first contact at lower tiers of the health system.
- Conceptual training alone is unlikely to engender this improvement, as knowledge among village and township level providers, though low in absolute terms, is already high relative to clinical practice.

## Introduction

National prevalence surveys conducted by the Chinese Center for Disease Control and Prevention (CCDC) show that between 1990 and 2010 smear-positive prevalence of tuberculosis (TB) decreased by 65%, from 170 to 59 per 100,000 [1]. Though improved socioeconomic conditions almost certainly helped, better treatment of those diagnosed with TB is also thought to have played an important role, as evidenced by large reductions in the number of smear-positive cases among previously diagnosed individuals. In part due to this success, by 2010 the large majority (approximately 90%) of smear-positive cases were individuals who had not received a previous diagnosis of TB. This suggests that further progress in China—which remains a high-burden country second only to India—will rely on improving case detection, most importantly in rural areas where the prevalence of TB is 3 times the national average at 163 per 100,000 and patients report longer diagnostic and treatment delays compared to urban patients [2–3].

Case detection in China’s rural areas already depends critically on the diagnostic ability of providers in village clinics (VCs) and township health centers (THCs), which are the bottom 2 tiers in the rural health system and the first contact for most rural TB patients [2]. Moving forward, the reliance on the lower tiers of the health system is only projected to increase as China moves towards an “integrated care” model that promotes initial contact with village and township providers and could potentially increase their gatekeeping role within the system [4,5]. Given the importance of the lower tiers in addressing rural TB, it is therefore surprising that, despite considerable speculation and some data on structural measures of quality, we currently have no information on how presumptive TB patients are diagnosed and treated when they visit grassroots primary care providers [4,6].

Our study therefore has 3 aims. First, we assessed the ability of primary care providers in VCs, THCs, and county hospitals to correctly diagnose and manage a presumptive TB patient. In order to do so, we evaluated the treatment that standardized patients (SPs) received against international and national benchmarks for correct diagnosis and management. We defined correct management as referral to an upper level provider, recommendation for a chest X-ray (CXR), or recommendation for sputum testing. SPs—people recruited from the local community and extensively trained to present the same condition to multiple providers—are often regarded as the “gold standard” for measurement of clinical practice and have recently been validated in a similar setting for TB [7]. SPs have been shown to be less subject to bias than approaches relying on recall-based patient surveys and chart abstraction [7]. Moreover, SPs enable comparisons across providers because cases are predetermined with the same standardized presentation for all providers.

Second, we assessed the same providers in their knowledge of correct procedures through clinical vignettes. Comparing vignette results with those from SPs allows us to identify the gap between provider knowledge and practice [7,8] and therefore whether low levels of provider knowledge are a major constraint on the quality of TB detection and care.

The third aim was to develop a method that could better integrate quality of care at different levels of the health system with the care-seeking behavior of households. Taking into account the 3-tiered VC-THC-County Hospital system in China, we evaluated TB management at the system level accounting for provider referrals and performance at different tiers of the healthcare system. For instance, among patients who first visited the VC, the system would have correctly managed the patient either through (a) correct management at the VC or (b) referral from the VC to the THC and correct management at the THC or (c) referral from the VC to the THC to the county hospital and correct management at the county hospital. Failure to either refer or correctly manage the patient at any point in the chain would have resulted in diagnostic failure.

The experience of real patients then depends on where they first choose to seek care. For instance, patients with prolonged cough and fever may “bypass” VCs and directly visit THCs or county hospitals as has been found in Tanzania and India [9,10]. We therefore weighted the provider-level management outcomes with the choices of real patients using supplementary data from a nationally-representative rural household survey and computed system-level correct case management proportions from the patient perspective. We then simulated system-level outcomes under alternative policies, such as what would happen if patients could visit THCs only after receiving a referral from the VC. The goal of these simulations was to estimate the potential effects of reforms encouraging initial contact with providers in VCs and THCs; these simulations assumed that provider quality remains fixed at the current levels.

## Methods

### Ethical approval

Approvals from the institutional review boards of Stanford University, USA (protocol number: 25904) and Sichuan University, China (protocol number: K2015025) were obtained. Informed consent was obtained verbally from all providers participating in the study. To prevent influence on the study, both IRBs approved a procedure whereby providers consented to SP visits “at some point in the next six months.” Consent from village and township providers was obtained as part of the facility survey approximately 5 weeks before SP visits. Consent for county providers was obtained through communications with providers. All individuals who participated as SPs were trained to protect themselves from any invasive tests or procedures.

### Setting and study design

China’s rural health system is comprised of 3 tiers of providers: VCs, THCs, and county hospitals. Under existing guidelines [11], VCs and THCs are responsible for referring suspected TB patients to higher-level providers (at the county level and above) for further diagnosis and care, although patients are free to choose among any of the 3 tiers for primary care without referral. Prior to 2011, suspected TB patients were to be referred from the regular health system to separate county-level facilities under the CCDC that diagnosed and treated TB patients following the Directly Observed Therapy, short course (DOTs) strategy [11]. Since then, however, these functions have been shifted to county-level hospitals within the regular health system.

With this shift of ultimate responsibility for diagnosis and treatment from the CCDC system to the regular health system, further success of TB control in China will likely become tied to the quality of care and success of several ongoing reforms in the health system. For instance, since the early 2000s, China has dramatically expanded coverage under public health insurance schemes, which is now close to universal in rural areas. Available evidence suggests that expanding coverage increased utilization, but did not reduce out-of-pocket expenses [12]. In 2009, the government then announced a series of reforms to address issues related to perceived inefficiency and fragmentation in the health system, including “perverse” physician incentives tied to drug sales and overcrowding at upper levels [4,5]. While implementation varies widely across health systems, these reforms are aimed at strengthening primary care and moving toward an integrated care model that encourages initial contact with village and township level providers. Although, as of March 2017, the specific design of these policies and the balance between gatekeeping by primary care providers versus patient choice still remains under consideration, encouraging initial contact with lower-tier providers could be consequential for progress against TB if the performance of these providers acts as a barrier to proper diagnosis and/or referral of TB patients.

### Selection of facilities, data collection, and study size

The results presented here are part of a larger cross-sectional study on quality of care in rural China. The sample for this study was drawn from rural areas in 3 provinces: Sichuan, Shaanxi, and Anhui, which are located in western, central, and eastern China, respectively. These 3 provinces have an overall prevalence of TB (urban and rural combined) on par with the national average (around 60 to 70 per 100,000 in Sichuan, 57 in Shaanxi, and 62 in Anhui) [13].

The 279 providers included in the study were selected from 1 prefecture (the administrative level below the province and above the county) in each of the 3 provinces, out of a total of 47 prefectures (10 in Shaanxi, 21 in Sichuan, and 16 in Anhui). The prefectures included in the study from each province were chosen for having a predominantly rural population in consultation with local authorities.

The sample was then selected to be representative of rural health systems (triplets, or referral chains, of village, township, and county-level health facilities) in each of the 3 chosen prefectures. We used the following procedure to sample health systems. First, across the 3 prefectures, we randomly sampled 21 of 24 rural counties and included the primary county-level hospital in each sampled county. Next, 10 THCs were randomly sampled within each county. Because even counties designated as “rural” have an urban township housing the county seat, we excluded the health center of the urban township. One county only had 9 rural townships, yielding a sample of 209 of the total 311 THCs in the 21 sample counties. Finally, we randomly selected one VC associated with each sampled THC for a total of 209 VCs. Out of the 209 originally-sampled villages, 22% had no VC and were replaced with a randomly-chosen backup. The health systems represented by this sample serve a population of 12.23 million people [14].

**Fig 1:**
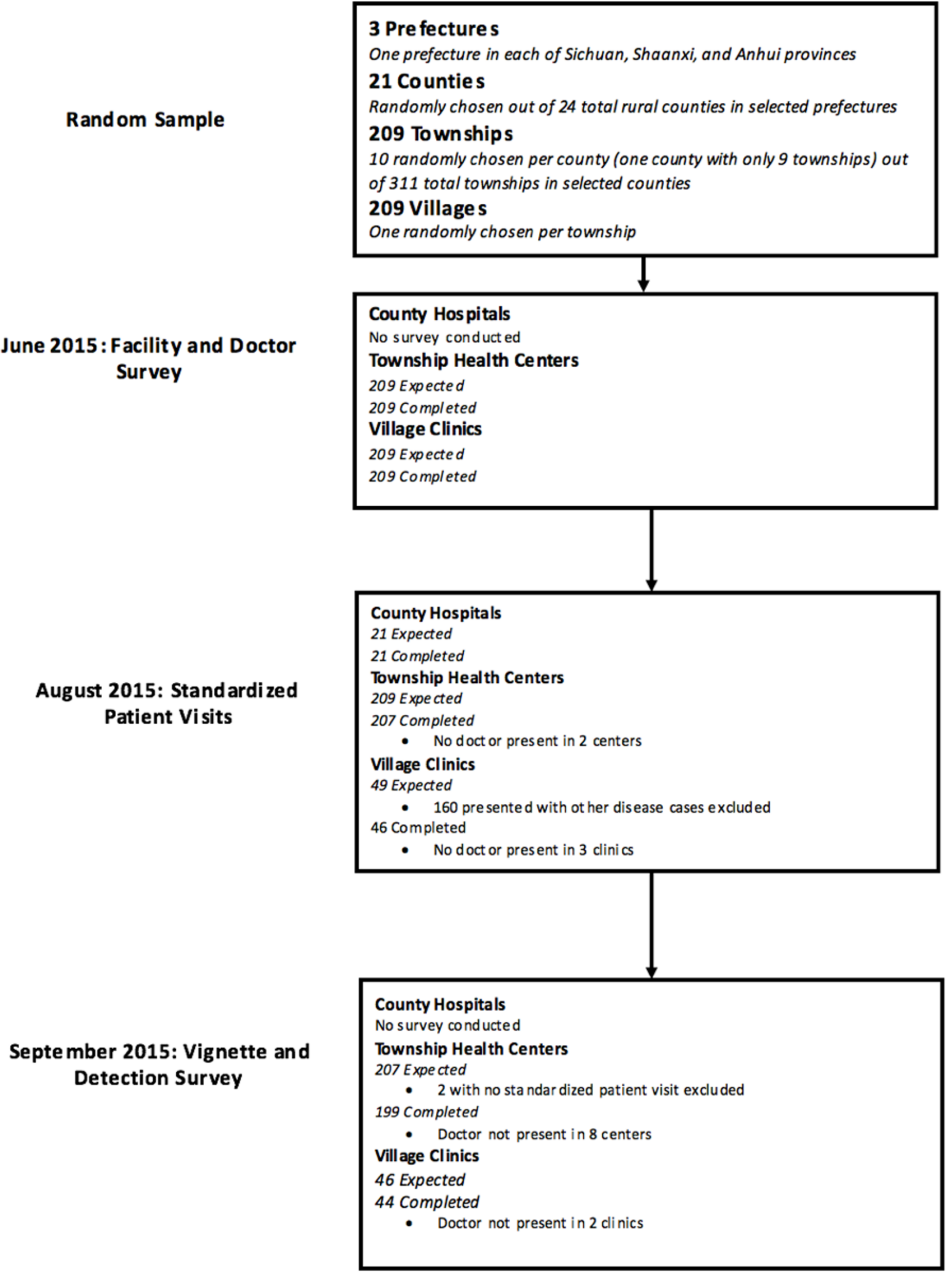
STROBE flowchart. SPs were randomly assigned to facilities and within each facility, SPs visited the doctor following the normal procedures for any walk-in patient. Given a choice of which doctor to visit, SPs randomly chose a doctor following a pre-determined randomization protocol. In county hospitals, where patients can choose doctors by specialty, SPs visited generalists. Our results, therefore, are designed to approach the care a walk-in patient would receive at each of the sampled facilities.

We conducted 3 separate waves of data collection (Fig 1). An initial facility and doctor survey was conducted for village and township level providers (but not county hospitals) in June 2015. Actual SP visits started 5 weeks after the initial facility survey in August 2015. SPs presenting symptoms of TB visited doctors in all sampled county hospitals and THCs. At the village level, however, SPs presenting with TB were only sent to a random subset of 49 VCs. This was because the larger study included SPs presenting other disease conditions and sending more than 1 SP to VCs would have significantly increased the risk that SPs would be identified as fake patients. Therefore, our sample contains 49 complete health systems (triplets of village, township, and county level providers) and 209 township and county level duplets.

Finally, we conducted a follow-up survey with village and township providers in early September 2015. In this survey, we asked providers whether they had detected any SPs and administered vignettes. S1 Text discusses the details of sample selection, assignment of SPs to providers, administration of surveys and vignettes and drug identification.

#### SPs

The SP case used in this study was adapted to the Chinese context from an earlier validation study in India in collaboration with TB experts from the China TB-CDC [7]. In this presumptive TB case, the SP presents with a “cough that is not getting better, and a fever.” Upon appropriate questioning by the provider, the SP reveals symptoms consistent with a classic case of presumed TB including a cough duration of 2–3 weeks, fever with night sweats, and loss of appetite and weight. Twenty-one SPs (10 male, 11 female) were recruited from local areas and intensively trained over a 2-week period to consistently present the case to providers in the sample.

Following the interaction, SPs purchased all medications prescribed and paid providers their usual fee. After each visit, SPs were debriefed using a structured questionnaire and SP responses were confirmed against a recording of the interaction taken using a concealed recording device. S1 Text provides further details and a link to the SP case script (in Mandarin with an English translation).

#### Clinical vignettes

In September 2015, we tested the sampled village and township providers using clinical vignettes for the same case presentation. Vignettes were administered by two enumerators, one playing the role of the patient and the other providing instructions and recording the interaction. At the start, providers were asked to proceed as they would with a real patient and were told that the patient would answer any questions and comply with any instructions. We use this vignette to assess provider knowledge of diagnosis and treatment and compare their performance on the vignette to what they did in practice with the SP. This difference between what providers know to do and what they do in practice is referred to as the “know-do gap.”

#### Supplementary household survey data on patient sorting

Part of our analysis requires information on how individuals select into the tiers of the rural health system given the symptoms described in the SP script. We obtained this information by including questions in a nationally-representative survey of rural households conducted in April 2016. Specifically, we asked a sample of 2,022 heads of households about healthcare-seeking behavior given a cough and fever lasting for 2 weeks. We asked both hypothetical and retrospective versions of these questions. Details on this survey are in S1 Text.

### Outcomes

Whether the case was correctly managed and evaluated was assessed in comparison to national standards for TB Care in China and the International Standards for TB Care, 3rd Edition [15–17]. We consider “Correct Management” to be referral (either verbal or written) to an upper level health system provider or CCDC facility, recommendation for a CXR, or recommendation for further sputum testing for TB (i.e., smear microscopy, PCR, culture). This is a lenient definition of correct management for 2 reasons. First, unnecessary and/or harmful medicines are not penalized—a stricter definition would exclude from correct management those cases where additional (unnecessary or even harmful) medicines were given, reducing the proportion of correct management by a significant margin. However, we report the use of (unnecessary) antibiotics, fluoroquinolones, and steroids. Antimicrobial resistance is a severe public health concern in China [18–20] while fluoroquinolones tend to mask underlying TB and steroids to aggravate latent or subclinical TB [21]. Second, the definition is particularly lenient at the level of the county hospitals, since a CXR is classified as correct management in our scheme, while such hospitals can be reasonably expected to move beyond CXR screening to order confirmatory microbiological sputum tests.

In addition to whether SPs were correctly managed, we also assess the providers’ adherence to a prespecified checklist of recommended questions and examinations. We also indicate a short list of “essential” items according to experts from the China TB-CDC. Further details on these study outcomes are in S1 Text.

### Statistical analysis

We calculate the proportion or mean and 95% CI by facility level (village, township, and county) for correct case management and its individual components (referral, CXR, or sputum test) as well as for the use of antibiotics, fluoroquinolones, and steroids and the number and percent of checklist items completed.

To assess the difference in case management between different provider tiers and SP and vignette interactions, we used Ordinary Least Squares (OLS) regressions for continuous dependent variables and logistic regressions for binary dependent variables with indicator variables for each county as additional controls. We also used logistic regressions to assess associations between VC and THC provider characteristics and case management outcomes, controlling for county and SP dummy variables. We report these results as odds ratios with accompanying 95% CI.

A novelty of our approach is the ability to construct system-level results for TB care. To do so, we first define a system-level observation as correctly managed if SPs are eventually referred to a provider who recommends a CXR or sputum test or refers the SP to a CCDC facility. For instance, if the SP visits a village provider and is told to visit the THC, we would then incorporate the results from the THC visit to simulate the entire referral chain in the entire system. Note that the systems-level outcome will be incorrect if there is a failure at any point in the referral chain.

We simulate system-level results under 3 alternative “managed referral” policies which vary in how the initial provider visited in the system is selected. First, we assume that patients are free to select their initial provider (the status quo). Here, we use probabilities that patients select providers at each level from the nationally-representative rural household survey. We then present 2 sets of results, assuming patients start at the village level (i.e., there is gatekeeping from the village level) and assuming patients start at the township level (i.e., there is gatekeeping from the township level).

For each of these 3 “managed referral” policies (status quo, village-level gatekeeping, and township-level gatekeeping), we simulate system-level results assuming that referrals to higher tiers in the health system happen in 1 of 2 ways. First, we assume that if SPs are referred by a provider, patients are assumed to go to the next level above the referring provider. Specifically, if an SP was referred by a village provider, the assessment progresses to the township provider above that village. If a township provider refers the SP, assessment progresses to the county hospital above that THC. Second, we simulate system-level results assuming that, upon referral, the assessment progresses to the provider that the SP was referred to. If a village provider refers the SP directly to the county hospital, the township is skipped and assessment progresses directly to the county. If patients are referred to the CCDC or above the county, we count the case as correctly managed.

Our primary system level results use data from the random subsample of 49 “complete” health systems. Results using all 209 health systems—but imputing referral rates for VCs in remaining systems without VC observations using average rates from observed VCs—are shown in S6 Table.

All analyses were done using Stata 14 (Stata Corporation, College Station, TX). A description of the timing of analysis decisions included in S1 Text and S1 STROBE Checklist provides the completed STROBE checklist.

## Results

### Basic description of facilities

Similar to previous findings in the literature [22], facility surveys confirmed the increasing qualifications of staff and better infrastructure at higher levels of care (S1 Table). For instance, although all providers are certified, no village providers had a “practicing physician” certificate, which is the highest of 3 levels above “assistant practicing physician” and “rural physician” certifications, compared to 61% of township providers. Similarly, 9% of village doctors had at least an upper secondary degree compared to 60% of township doctors. Finally, no VCs had X-ray machines or the ability to conduct smear microscopy. In contrast, 89% of THCs had an X-ray machine, although only 56% had staff able to operate an X-ray machine, and only 2% could conduct smear microscopy. Although not directly measured, county hospitals generally have X-ray machines as well as the ability to conduct smear microscopy [23]. County-level doctors tend to have higher education and certification levels than those in THCs.

### SP sample completion rates

SP visits were successfully completed in 274 of 279 of the sampled facilities (46 VCs, 207 THCs, and 21 county hospitals). As shown in Fig 1, no doctors were present in 3 sampled VCs and 2 sampled THCs at the time of SP visits. No providers declined visits from SPs. Of the 253 village and township doctors visited by SPs, 243 completed the vignette and detection survey. Of these, 9 of 243 (4%, 95% CI 2%–7%) reported that they suspected someone as an SP, while physician descriptions matched the SP in 6 interactions (3%, 95% CI 1%–5%). As no provider voiced suspicion during the interaction, these detections may have occurred because SPs did not return with test results that had been requested and will not affect the interpretation of the data presented here.

### Management of SPs

On average, interactions between SPs and providers were brief, lasting 11 mins on average, though patient loads in village and THCs were low (Table 1). Overall, 111 of 274 SPs (41%, 95% CI 35%–46%) were correctly managed across all tiers of the health system (Table 1). Where interactions were correctly managed, this was either because the provider referred the patient or suggested radiological testing; microbiological testing was infrequently used (10 of 274 cases). Though not considered incorrect in our lenient definition of correct management, doctors frequently prescribed broad-spectrum antibiotics unrelated to the treatment of TB (168 of 274 interactions). The most commonly prescribed antibiotics were Macrolides, Penicillins, and Cephlasporins. Notably, quinolone and steroid use was low. TB drugs are controlled in China, and there were no cases where anti-TB treatment was initiated for an SP.

**Table 1:** Main outcomes of interactions with SPs.

	Full Sample	VCs	THCs	County Hospitals (CHs)	*P* value:	*P* value:	*P* value:
					VC versus THC	THC versus CH	VC versus CH
**Patient–provider interactions**		46	207	21			
**Patient load (number of patients waiting at time of SP visit)**		0.52 (0.14–0.91)	1.13 (0.83–1.42)	—	0.07	—	—
**Case management**	** **	** **					
**Correctly managed the case §**	41	28	38	90	0.388	<0.001	<0.001
	(35–46)	(17–43)	(32–45)	(71–97)			
** Ordered a chest radiograph**	36	17	35	90	0.049	<0.001	<0.001
	(31–42)	(9–31)	(29–42)	(71–97)			
** Ordered a sputum smear test**	4	0	4	5	0.150	0.935	0.136
	(2–7)	(0–8)	(2–8)	(1–23)			
** Referred to other providers**	19	28	18	5	0.113	0.135	0.067
	(14–24)	(17–43)	(13–24)	(1–23)			
** Referred to CCDC or DOTs, if referral**	29	8	38	0	0.090	0.219	0.496
	(19–43)	(1–33)	(24–54)	--			
** Referred to city provider, if referral**	10	8	11	0	0.766	0.520	0.496
	(4–21)	(1–33)	(4–25)	--			
** Referred to county provider, if referral**	51	46	51	1	0.933	0.495	0.067
	(38–64)	(23–71)	(36–67)	(21–100)			
** Referred to town provider, if referral**	10	38	0	0	0.039	—	0.116
	(4–21)	(18–64)	—	—			
**Asked patient to return**	11	11	11	19	0.763	0.234	0.180
	(8–16)	(5–23)	(7–16)	(8–40)			
**Gave antibiotics and steroid**	4	0	6	0	0.094	0.094	—
	(3–7)	—	(3–10)	—			
**Gave any antibiotic**	61	63	65	24	0.773	0.001	0.003
	(55–67)	(49–75)	(58–71)	(11–45)			
**Gave any fluoroquinolone**	7	2	8	0	0.205	0.172	0.496
	(4–10)	(0–11)	(5–13)	—			
**Gave any steroid**	5	0	7	0	0.071	0.219	—
	(3–8)	—	(4–11)	—			
**Process**							
**Time with provider (min)**	11.43	10.18	12.09	7.67	0.149	0.01	0.370
	(10.56–12.31)	(8.39–11.97)	(11.08–13.11)	(3.96–11.37)			
**Number of questions and examinations (ISTC)**	5.56	4.50	5.90	4.57	0.001	0.019	0.926
	(5.26–5.86)	(3.73–5.27)	(5.56–6.24)	(3.58–5.56)			
**% of questions and examinations (ISTC)**	18	15	19	15	0.001	0.019	0.926
	(17–19)	(12–17)	(18–20)	(12–18)			
**Number of questions and examinations (China)**	3.98	3.11	4.19	3.76	0.001	0.373	0.124
	(3.73–4.23)	(2.5–3.71)	(3.9–4.48)	(2.97–4.55)			
**% of questions and examinations (China)**	22	17	23	21	0.001	0.373	0.124
	(21–23)	(14–21)	(22–25)	(17–25)			
**% of essential history checklist asked by provider (Both Standards)**	35	26	38	31	<0.001	0.096	0.544
	(33–38)	(20–32)	(35–40)	(23–39)			
**Cost of consultation and medicines combined (Chinese Yuan)**	27.18	23.45	28.01	27.19	0.266	0.898	0.640
	(23.52–30.84)	(17.07–29.82)	(23.76–32.26)	(7.29–47.09)			
Cost of consultation and medicines combined (US dollars)*	4.18	3.61	4.31	4.18	0.266	0.898	0.640
	(3.62–4.74)	(2.63–4.59)	(3.66–4.96)	(1.12–7.24)			
**Diagnosis**							
**Mentioned TB**	15	04	15	29	0.071	0.112	0.008
	(11–19)	(1–15)	(11–21)	(14–50)			

CCDC, Chinese Center for Disease Control and Prevention; CH, county hopsitals; DOTs, Directly Observed Therapy, short course; ISTC, international standards for tuberculosis care; SP, standardized patient; TB, tuberculosis; THC, township health center; VC, village clinic

Data are % (95% CI) unless otherwise noted.

*$1 = CNY6.5.

§Correctly managed for Tuberculosis is defined as a chest radiograph, sputum test, or referral. "ISTC" is ISTC standard and "China" is China national standard.

**Fig 2:**
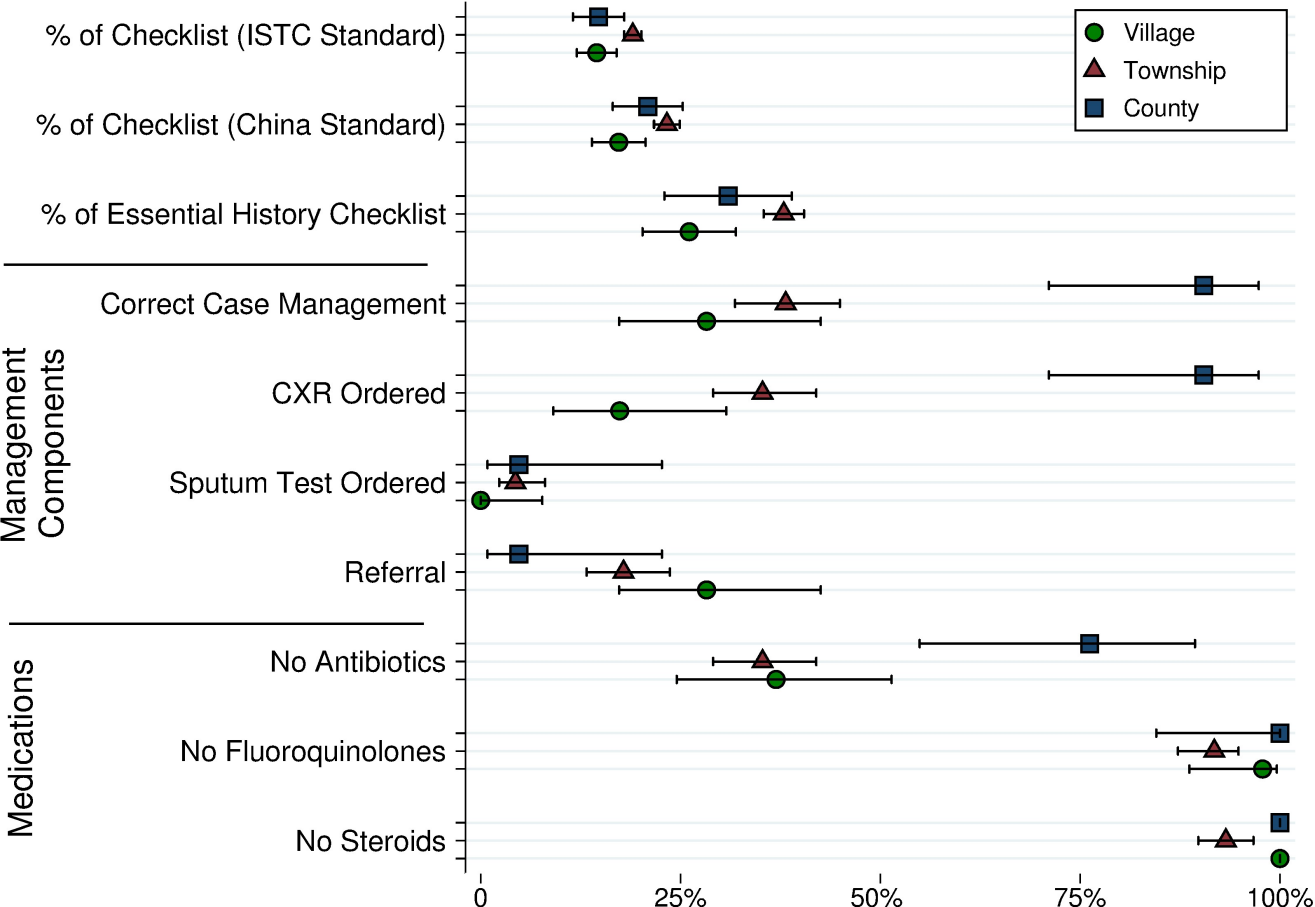
Main outcomes of interactions with SPs. Presaging our simulation results, in the 13 of 46 cases (28%, 95% CI 17%–43%) when village providers (verbally) referred SPs, 5 of 13 (38%, 95% CI 18%–64%) referred to the THC and 6 of 13 (46%, 95% CI 23%–71%) referred directly to the county. Of the 36 (18%, 95% CI 13%–24%) township providers who referred SPs to an upper level provider, a small majority referred to the county hospital (18 of 36; 51%, 95% CI 36%–67%), and the rest referred directly to the CCDC (14 of 36; 39%, 95% CI 25%–55%) or a city level provider (4 of 36; 11%, 95% CI 4%–25%). CXR, chest X-ray; ISTC, international standards for tuberculosis care; SP, standardized patient; THC, township health center.

Case management improved at higher levels of the health system, with SPs correctly managed in 19 of 21 (90%, 95% CI 71%–97%) interactions with doctors in county hospitals compared to 79 of 207 (38%, 95% CI 32%–45%) interactions with THC doctors and 13 of 46 (28%, 95% CI 17%–43%) interactions with village doctors (Table 1 and Fig 2). The proportion of correctly managed interactions was significantly higher in county hospitals than in VCs (OR 0.02, 95% CI: 0.0–0.17, *p* < 0.001) and THCs (OR 0.06, 95% CI: 0.01–0.25, *p* < 0.001), primarily due to the greater use of chest radiography. If county hospitals were held to a higher standard of requiring sputum TB testing of the SPs, only 5% would have met that standard. Similarly, the use of non-TB antibiotics was significantly lower in county hospitals (county versus village: *p* = 0.003; county versus township: *p* = 0.001), although it was above 60% in both VCs and THCs. Finally, while negligible in VCs and county hospitals, 17 (8%, 95% CI 5%–13%) THC doctors prescribed fluoroquinolones and 14 (7%, 95% CI 4%–11%) prescribed steroids.

Table 1 and Fig 2 also report adherence to a prespecified checklist of recommended questions and examinations. Completion rates for each checklist item are shown in S2 Table. S1 Fig shows patient outcomes by diagnosis for each health system tier.

**Fig 3:**
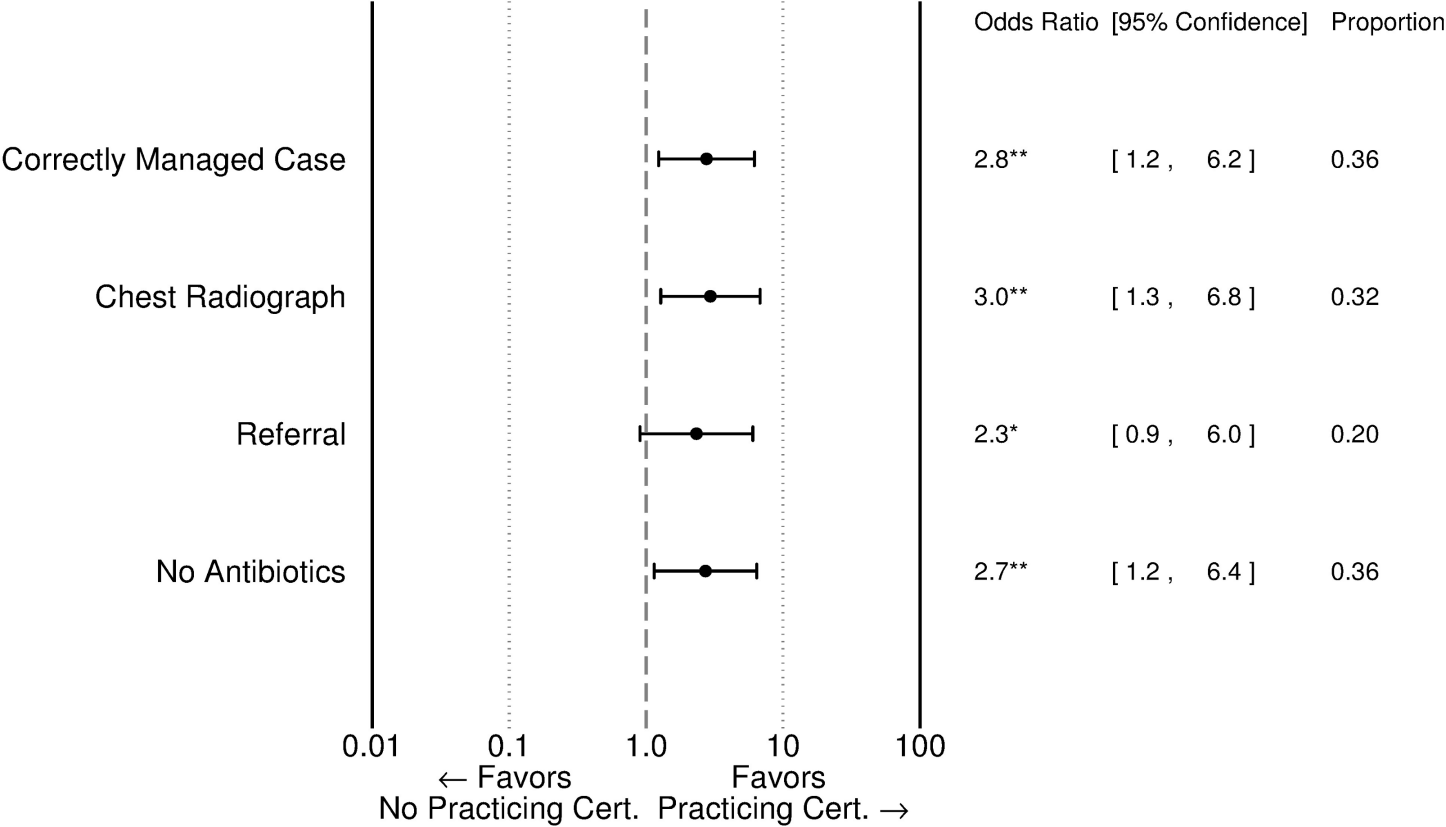
Correlation between provider certification and SP outcomes among village and township providers. Odds ratios are adjusted for additional facility and doctor characteristics using regressions reported in S4 Table. A “Practicing Physician” certification is the highest of 3 certification levels above “Assistant Practicing Physician” and “Rural Physician” certifications. “Proportion” refers to the proportion in the full sample of village and township providers. * *p* < 0.10, ** *p* < 0.05, *** *p* <0.01. SP, standardized patient.

In part, quality variation by level of care reflects differences in the characteristics of providers. Fig 3 and S3 Table show that providers at the village and township levels with a practicing physician certificate were significantly more likely to correctly manage TB cases than those with lower qualifications; more likely to request a CXR; more likely to refer patients to upper levels; and less likely to prescribe antibiotics. Correct management, request of chest radiographs, and prescription of antibiotics were also highly associated with whether facilities had professional staff on site able to operate X-ray machines. We find that the correct management of SPs in THCs with X-ray equipment and staff was 18 percentage points higher (53 of 115 with equipment and staff compared to 26 of 92 without).

### Knowledge versus practice deficits—The “know-do” gap

**Fig 4:**
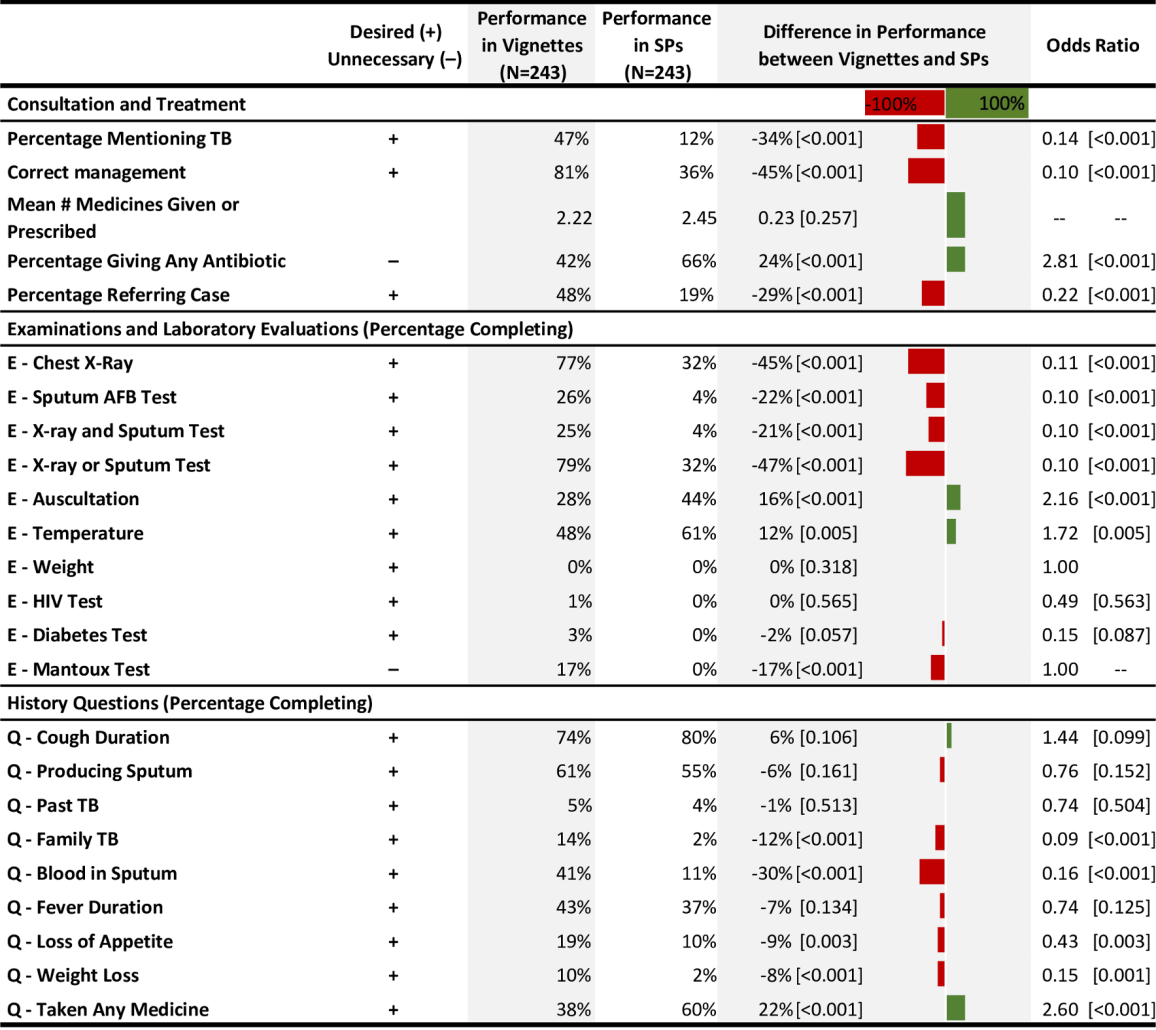
Know-do gap: Comparison of data from vignettes versus SP interaction among the same providers. For all items, the prefix "E" indicates examinations and laboratory evaluations; the prefix "Q" indicates history questions. The gap calculation is the result of a *t* test comparing the average vignette performance with the average SP performance. *P* values are in brackets. AFB, acid-fast bacillus; SP, standardized patient; TB, tuberculosis.

In direct measurements of knowledge using vignettes among village and township doctors, 81% of cases were correctly managed (Fig 4). This proportion of cases correctly managed was 45 percentage points (*p* < 0.001; 95% CI 37%–53%) higher than in SP interactions, reflecting the significantly higher use of chest radiographs and sputum testing as well as the increased willingness to refer in the vignettes. Providers were also less likely to prescribe antibiotics in the vignettes.

### Simulations of managed referral policies

In the household survey, 97% of respondents indicated that they would see a doctor if a family member had a cough and fever lasting for 2 weeks (S5 Table). Of these, 46% indicated that they would first visit their VC, 31% indicated they would first visit their THC, and 23% indicated they would directly visit a county or higher-level hospital. These percentages are consistent with responses of the 16% of households with a member who actually experienced these symptoms in 2015 (S5 Table, Panel B).

**Fig 5:**
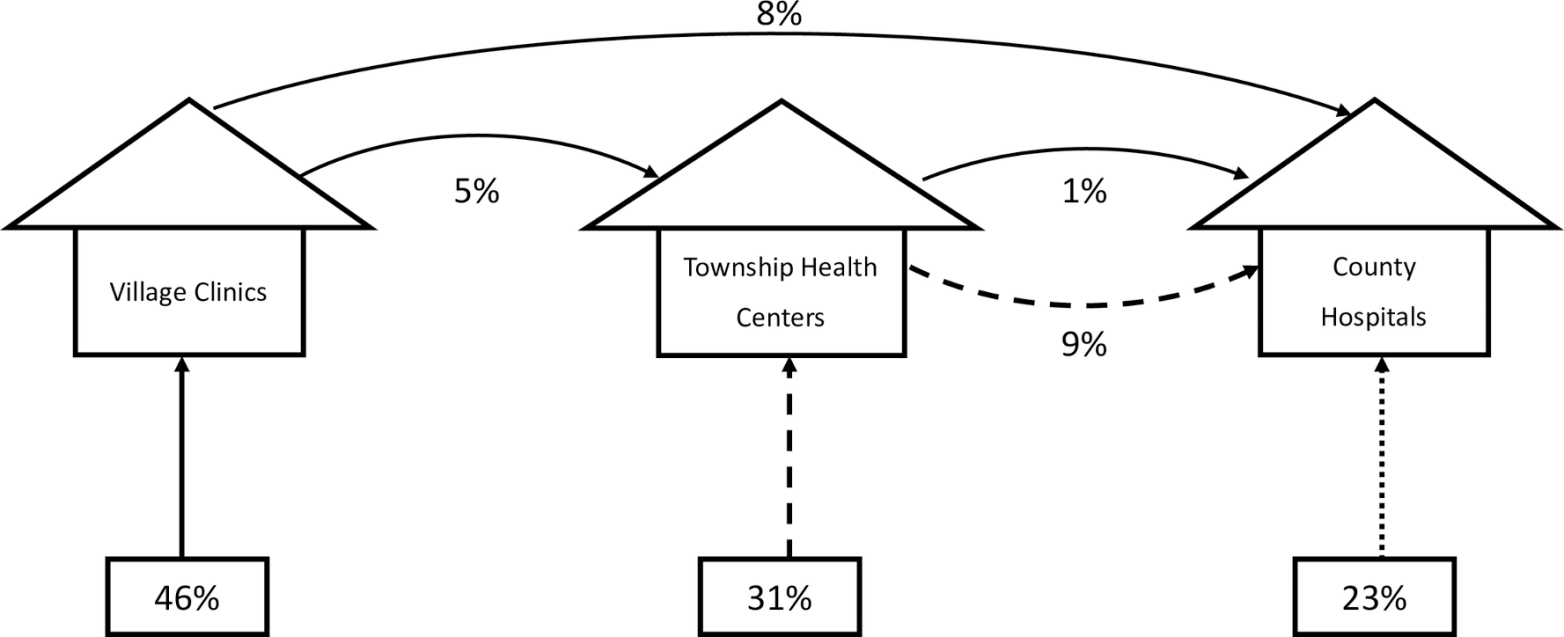
Estimated patient pathways under status quo (patients freely selecting into tiers). Percentages at bottom of figure show the percentage of patients selecting into each health system tier based on survey responses (S5 Table). For each referral pathway, figure shows percentage of total patient population following each path calculated using SP results for subsample of complete health systems.

We use these responses together with our results on case management at each level of the health system to generate “system level quality” under the status quo (patients freely selecting into tiers) and different managed referral policies. Fig 5 shows what happens under the status quo. In this case, 46% of patients would visit the VC. We know from our SP results that 28% of these cases (13% of all patients) would be referred and, of those referred, 38% (5% of all patients) would be referred to the THC and remainder to county or higher. In the subsample of full systems, of 31% of patients choosing to first visit the THC, 29% (9% of all patients) of patients would be referred to the county level or above. Finally, 24% would go directly to the county. This “cascade” of care allows us to compute the rate of correct management at the system level.

Under the status quo, we estimate that 34% (95% CI 27%–41%) of cases (where patients visit a provider) would be correctly managed at the system level if referrals were only allowed to progress to the next tier (Table 2). Instead, if we follow the actual referrals, which often bypassed the next tier, the correct management rate increases to 40% (95% CI 34%–47%). Therefore, referral behavior appears to account for deficits in the care at different tiers of the health system.

**Table 2:** Simulation of system-level management outcomes with and without managed referrals.

	Patients Select Initial Provider Level*	Managed Referrals^§^	
		Start from VC	Start from THC
% Correctly Managed with Straight Referrals^☨^	34% (27–41)	4% (1–15)	35% (28–42)
% Correctly Managed with True Referrals	40% (34–47)	16% (7–30)	37% (30–44)

THC, township health center; VC, village clinic

Data are % (95% CI).

*Patient sorting in 'Patients Select Initial Provider Level' column based on a nationally representative sample of rural households: 45.7% at village, 30.87% at township, 23.43% at county.

^§^Managed referrals refer to patients being required to initially visit providers at the village or township level.

^☨^“Straight Referrals” refers to referrals only being allowed to the next highest tier. “True Referrals” allow for bypassing.

S2 Fig simulates different managed referral policies with results summarized in Table 2. One possibility is that gatekeeping occurs at the level of the VC. In such a case, patients would be required to obtain a referral from the VC. We simulate that under this policy only 4% (95% CI 1%–15%) of cases would be correctly managed if referrals were only allowed to the next highest tier and would be 16% (95% CI 7%–30%) if the patterns of referrals allowed for bypassing and followed the same pattern we observe in our data. A second alternative is that gatekeeping is at the level of the THC. In this case, simulations of managed referrals from the township produce results on par with the status quo.

S6 Table shows simulation results using all 209 health systems, but imputing referral rates for VCs in remaining systems without VC observations using average rates from observed VCs. S7 Table shows simulated out-of-pocket costs for each scenario presented above; however, it should be noted that these estimates do not fully account for patient costs (such as indirect costs incurred by patients to travel to higher-tier facilities) that are likely important determinants of relative cost-effectiveness of the different managed referral policies.

## Discussion

Our study uses SPs to evaluate the management of TB among healthcare providers in China. We find that rates of correct management are low among village and township level providers in rural areas. Given that most rural patients with TB symptoms initially see providers at these levels, our results suggest that deficits in performance can contribute significantly to delayed detection of TB in China. The poor quality of care that we found for TB is consistent with previous findings for other conditions in studies using SPs [6]. Like in other studies, even in those cases that are correctly managed, there is a preference for radiography over microbiological testing. This contrasts with WHO recommendations for TB diagnosis, as CXRs often yield significant false positives.

Deficits in care only partially reflect poor knowledge of how to diagnose and manage TB cases. Correct case management proportions are significantly higher in the vignettes than with the SPs, suggesting a sizeable gap between physician knowledge and practice. This “know-do” gap has been reported in every comparison of vignettes and SPs and highlights the considerable discordance between national and international guidelines of TB care and the actual experience of patients [23–26]. Although policymakers recognize that guidelines tend to be aspirational, the study highlights the scale of the discordance between guidelines and practice. There are 3 classes of explanation for why this discordance arises.

The first has to do with “treatment that is best for the patient, but not for society.” Most patients with these symptoms will likely not have TB. Therefore, even though the correct actions can reduce transmission, providers may prefer a “wait and see” approach, using a cocktail of broad spectrum antibiotics and evaluating the patient if he/she does not improve. Patients may prefer this approach, as they do not have to undergo time-intensive tests and the psychological consequences of a potential TB diagnosis. It is worth highlighting though, that providers do not appear to follow a set protocol that is based on reasonable and pragmatic norms recognized as best practice in given settings. We see considerable variation in the way SPs are treated, ranging from the use of antibiotics and quinolones to the times spent and questions that are asked. Similar variation has been found in Delhi, India, but not in Nairobi, Kenya, where half of all SP visits to the public sector for the same case that we used here resulted in a recommendation for a sputum test [27].

The second class of explanations recognizes that some actions benefit the provider at the expense of the patient’s own welfare, due to weak or misaligned incentives. The link between provider compensation and drug sales [28], for instance, implies that providers have an incentive to “retain” rather than refer their patients [29] and—worryingly, given China’s high incidence of drug-resistant tuberculosis (DR-TB) [30]—use antibiotics inappropriately.

Finally, a third class of explanations assume that providers indeed wish to follow protocols, but are hampered by the lack of necessary equipment or very high patient loads. As to the first, we indeed find that the correct management of SPs in THCs with X-ray equipment and staff was 18 percentage points higher. However, even if this relationship were causal, it would still leave sizable deficits relative to standards of TB care. Moreover, providers could always refer patients to upper-level facilities for diagnostic testing but often fail to do so. As to high patient loads as a constraining factor, in sharp contrast to the view that providers have too many patients, we found only 0.52 (in VCs) and 1.13 (THCs) patients waiting when the SP visited. Healthcare providers spend 10 to 12 minutes with each SP despite considerable slack in their workdays.

Simulations under alternative managed referral policies suggest that—given the current quality of care—encouraging initial contact with village providers would reduce overall rates of correct management relative to the status quo of patients being able to freely select into provider tiers. In all our simulations, greater freedom in referral policies—whether through patient or provider choice—improves proportions of correct management. The basic assumption under systems of triage—that providers will recognize when a case is “serious” and refer appropriately—is not consistent with our data. In other words, if patients were to follow instructions of policymakers to first seek care in lower levels, the probability that they would be misdiagnosed, improperly treated, or harmed is higher than if they bypass the lower levels of care. In fact, a significant fraction of individuals in rural areas indeed bypass VCs at considerable cost, even when their symptoms are not indicative of a serious condition. Future research should evaluate the relative efficiency of policies relying on patient or provider choice given differences in the quality of care for different diseases in the health system. The novel system level sampling that we implement here can be regularly incorporated into SP studies to provide ex-ante estimates of different gatekeeping policies in China and other countries.

### Limitations

There are several limitations to our study. First, we evaluate the management of a single SP depicting a classic case of presumptive TB. As a result, we do not know how physicians may deal with more complicated TB presentations (patients with recurrent TB symptoms, suggestive of drug-resistant TB) and whether physicians can correctly treat known cases of TB. Das et al. 2015 [7], for instance, evaluated physician management of 4 different cases, including a confirmed case of TB and suspected multi-drug resistant tuberculosis (MDR-TB).

A second limitation is that we evaluate one-time new patient interactions. Due to increased risk of detection, SPs did not complete follow up visits with the 11% of physicians who asked patients to return (see Table 1). Moreover, SPs were completely unknown to physicians; physician treatment of known, regular patients may differ. Differences due to this are more likely at the village level than with township and county level providers.

Third, SPs did not complete invasive tests, and it could be that the completion of these tests would have led to different treatment paths for real patients. We can therefore evaluate the treatment path only to the point that an invasive test was ordered. Particularly in the case of county hospitals, this is likely to have resulted in an overly-lenient assessment of case management since nearly all suggested chest radiographs, but we are unable to determine whether this was part of their work-up for TB or whether they routinely order CXR for all patients or for financial or other nonmedical reasons. However, in another exercise, we did present doctors in county hospitals with CXRs of actual TB patients provided by the CCDC chosen to match the case presented in the SP script. After being shown this X-ray, 32% of county-level doctors who ordered a CXR for SPs mentioned TB as a potential diagnosis, suggesting that the low use of microbiological testing persists even after an abnormal CXR.

Fourth, although SPs underwent intensive training aimed at standardizing their presentation of the disease case, there may nevertheless remain differences across SPs. Any differences across SPs however, are unlikely to significantly affect results, as SPs were randomly assigned to providers. Moreover, regression models including and excluding fixed effects for SPs yield similar coefficients on variables of interest. In models comparing village, township, and county differences, SP fixed effects explain only 7% of the overall variation in correct management in our data.

Our simulations of system-level outcomes under alternative managed referral policies also have a number of limitations. First, simulations assume that patients would follow suggested referrals. Second, these simulations hold patient and provider behavior fixed (by design). We do not consider how patients’ healthcare-seeking behavior or provider behavior may change as a result of mandated initial visits at lower levels of the health system. They also do not account for how providers at higher tiers may treat patients differently if patients were to reveal that they had been referred by lower-tier providers. Third, uncertainty in our simulations arise from sampling variation both in the nationally representative household survey and within the sample of providers visited by SPs. Fourth, there is imperfect overlap between the sampling frame of the nationally-representative rural household survey on patient selection of initial provider tiers used in simulations of management under the “status-quo” and the sampling frame for SP visits to providers.

Finally, our study sample was drawn to be representative of rural health systems in 3 chosen prefectures. Though we selected these prefectures to cover diverse regions (western, central, and eastern China) in consideration of geographical variation in health system quality, our sample is not nationally representative.

### Conclusion

Village and township providers in the rural health system perform poorly in diagnosis and management of a classic case of presumptive TB. Poor performance is due to not only a lack of provider knowledge but also a large gap between provider knowledge and practice. Given significant deficits in quality of care, reforms encouraging first contact with village providers in rural areas would undermine further progress against TB in China unless substantial efforts are also made to improve the management of patients with suspected TB in VCs and THCs. An effective approach could involve greater integration between health system tiers with mechanisms to facilitate the transfer of knowledge from higher to lower levels and strengthen incentives for appropriate referral but initially continuing to allow for greater patient choice.

## Supporting information

S1 STROBE checklist (PDF)

S1 Table Provider characteristics. (PDF)

S2 Table Completion of checklist items. (PDF)

S3 Table Correlates of correct management, CXR, referral, and antibiotic prescription of standardized patients (SPs) among village and township providers. (PDF)

S4 Table Additional statistics comparing vignettes and SP visits. (PDF)

S5 Table Patient selection into health system tiers with symptoms of TB. (PDF)

S6 Table Simulation of system-level management outcomes with and without managed referrals (all health systems). (PDF)

S7 Table Simulation of out-of-pocket costs with and without managed referrals. (PDF)

S1 Fig Case management of SPs. (PDF)

S2 Fig Estimated patient pathways under gatekeeping. (PDF)

S1 Text Methodological appendix. (PDF)

## Abbreviations

CCDC: Chinese Center for Disease Control and Prevention
CXR: chest X-ray
DOTs: Directly Observed Therapy, short course
DR-TB: drug-resistant tuberculosis
MDR-TB: multi-drug resistant tuberculosis
OLS: Ordinary Least Squares
SP: standardized patient
TB: tuberculosis
THC: township health center
VC: village clinic
