# Supplementary material for: Tuberculosis detection and the challenges of integrated care in rural China: A cross-sectional standardized patient study
Source: PLoS Med. 2017 Oct 17;14(10):e1002405. doi: 10.1371/journal.pmed.1002405 (PMC5644979; doi:10.1371/journal.pmed.1002405)
Supplement: S1 STROBE checklist — (PDF) [file pmed.1002405.s001.pdf]

STROBE Statement—Checklist of items that should be included in reports of ***cross-sectional studies***

	Item No	Recommendation
**Title and abstract**	1	(*a*) Indicate the study's design with a commonly used term in the title or the abstract *The title describes the study design as "a cross-sectional standardized patient study".*
		(*b*) Provide in the abstract an informative and balanced summary of what was done and what was found *The abstract describes the methods and findings.*
**Introduction**		
Background/rationale	2	Explain the scientific background and rationale for the investigation being reported *The background and rationale are described in the Introduction, paragraphs 1 & 2.*
Objectives	3	State specific objectives, including any prespecified hypotheses *The specific aims of the study are stated in the Introduction, paragraphs 3-5.*
**Methods**		
Study design	4	Present key elements of study design early in the paper *The study design is discussed in paragraphs 3-5 of the Introduction and paragraphs 3-12 of the Methods section.*
Setting	5	Describe the setting, locations, and relevant dates, including periods of recruitment, exposure, follow-up, and data collection *The institutional setting is described in paragraphs 1 and 2 of the Methods section; Study locations are described in paragraph 3 of the Methods section; and study timing is discussed in paragraphs 6-8 of the Methods section.*
Participants	6	(*a*) Give the eligibility criteria, and the sources and methods of selection of participants *Selection of the sample is discussed in paragraph 3 of the Methods section.*
Variables	7	Clearly define all outcomes, exposures, predictors, potential confounders, and effect modifiers. Give diagnostic criteria, if applicable *Outcomes are discussed in the Outcomes subsection (paragraphs 13 and 14 of the Methods section). The primary outcome of the study is whether cases were "correctly managed" defined as be referral (either verbal or written) to an upper level health system provider or the CDC, recommendation for a chest X-ray (CXR), or recommendation for further sputum testing for TB (i.e. smear microscopy, PCR, culture)."*
Data sources/ measurement	8*	For each variable of interest, give sources of data and details of methods of assessment (measurement). Describe comparability of assessment methods if there is more than one group *Measurement of the outcomes are discussed in the Outcomes subsection (paragraphs 13 and 14 of the Methods section).*
Bias	9	Describe any efforts to address potential sources of bias *The advantages of using standardized patients to measure clinical practice, including avoidance of common sources of bias inherent in other methods, are discussed in paragraph 3 if the Introduction.*
Study size	10	Explain how the study size was arrived at *Sample determination is discussed in paragraph 5 of the Methods section.*
Quantitative variables	11	Explain how quantitative variables were handled in the analyses. If applicable, describe which groupings were chosen and why

		Use of variables is discussed in the Statistical Analysis subsection.
Statistical methods	12	(*a*) Describe all statistical methods, including those used to control for confounding<br>*Statistical methods are discussed in the Statistical Analysis subsection.*
		(*b*) Describe any methods used to examine subgroups and interactions<br>*Described in paragraph 1 of the Statistical Analysis subsection. Results are analysed by facility level.*
		(*c*) Explain how missing data were addressed<br>*Missing observations are discussed in paragraph 3 of the Results and shown in the STROBE Flowchart. In the few cases where sampled providers did not receive SP visits, they were excluded from the analysis. Those receiving SP visits but not completing the vignette survey were excluded from analysis using vignettes. No individual variables are missing for providers included in the analysis.*
		(*d*) If applicable, describe analytical methods taking account of sampling strategy<br>*None.*
		(*e*) Describe any sensitivity analyses<br>*The next to last paragraph of the Statistical Analysis subsection discussed an additional analysis of system level results, shown in S5 Table, imputing referral rates for village clinics not visited by standardized patients.*
**Results**		
Participants	13*	(a) Report numbers of individuals at each stage of study—eg numbers potentially eligible, examined for eligibility, confirmed eligible, included in the study, completing follow-up, and analysed<br>*Data collection completion rates are discussed in paragraph 3 of the Results Section. The number of providers at each level completing each phase of data collection is also shown in the STROBE flowchart (S1 Fig).*
		(b) Give reasons for non-participation at each stage<br>*Discussed in paragraph 3 of the Results section and shown in the STROBE flowchart (S1 Fig).*
		(c) Consider use of a flow diagram<br>*S1 Fig. STROBE Flowchart.*
Descriptive data	14*	(a) Give characteristics of study participants (eg demographic, clinical, social) and information on exposures and potential confounders<br>*Provider characteristics are presented in S1 Table.*
		(b) Indicate number of participants with missing data for each variable of interest<br>*Presented in S1 Fig (STROBE Flowchart). There is no data missing from completed SP visits and vignettes.*
Outcome data	15*	Report numbers of outcome events or summary measures<br>*Both numbers and percentages/proportions are reported throughout the Results Section.*
Main results	16	(*a*) Give unadjusted estimates and, if applicable, confounder-adjusted estimates and their precision (eg, 95% confidence interval). Make clear which confounders were adjusted for and why they were included<br>*Unadjusted results are presented for all outcomes.*
		(*b*) Report category boundaries when continuous variables were categorized<br>*Not applicable.*
		(*c*) If relevant, consider translating estimates of relative risk into absolute risk for a meaningful time period

		Not applicable.
Other analyses	17	Report other analyses done—eg analyses of subgroups and interactions, and sensitivity analyses<br>*An additional analysis of system level results imputing referral rates for village clinics not visited by standardized patients shown in S5 Table.*
**Discussion**		
Key results	18	Summarise key results with reference to study objectives<br>*Results are summarized in paragraphs 1-3 of the Discussion section.*
Limitations	19	Discuss limitations of the study, taking into account sources of potential bias or imprecision. Discuss both direction and magnitude of any potential bias<br>*Limitations are discussed in paragraphs 4-8 of the Discussion section (Limitations subsection).*
Interpretation	20	Give a cautious overall interpretation of results considering objectives, limitations, multiplicity of analyses, results from similar studies, and other relevant evidence<br>*Final paragraph of Discussion section (Conclusion subsection).*
Generalisability	21	Discuss the generalisability (external validity) of the study results<br>*The representativeness of the sample is discussed in the final paragraph of the limitations subsection.*
**Other information**		
Funding	22	Give the source of funding and the role of the funders for the present study and, if applicable, for the original study on which the present article is based<br>*Metadata.*

*Give information separately for exposed and unexposed groups.

Note: An Explanation and Elaboration article discusses each checklist item and gives methodological background and published examples of transparent reporting. The STROBE checklist is best used in conjunction with this article (freely available on the Web sites of PLoS Medicine at http://www.plosmedicine.org/, Annals of Internal Medicine at http://www.annals.org/, and Epidemiology at http://www.epidem.com/). Information on the STROBE Initiative is available at www.strobe-statement.org.
