# Supplementary material for: Tuberculosis detection and the challenges of integrated care in rural China: A cross-sectional standardized patient study
Source: PLoS Med. 2017 Oct 17;14(10):e1002405. doi: 10.1371/journal.pmed.1002405 (PMC5644979; doi:10.1371/journal.pmed.1002405)
Supplement: S1 Table — (PDF) [file pmed.1002405.s002.pdf]

S1 Table. Provider Characteristics

Panel A: Facility Characteristics		
	**Village Clinics (N=46)**	**Township Health Centers (N=207)**
Number of patients in catchment area	2291 (1834-2747)	26184 (23380-28988)
Number of physicians working full time at the facility	1.52 (1.27-1.78)	7.68 (6.7-8.66)
Facility has X-Ray machine	0 (0%)	185 (89%, 84-93)
Facility has professional staff able to use X-ray machine	0 (0%)	115 (56%, 49-62)
Facility has sputum smear test equipment	0 (0%)	4 (2%, 1-5)
Facility has professional staff able to use sputum smear test equipment	0 (0%)	1 (0%, 0-3)
Facility has stethoscope	45 (98%, 89-100)	207 (100%, 98-100)
Facility has thermometer	46 (100%, 92-100)	207 (100%, 98-100)
Facility manages care of diagnosed TB patients	36 (78%, 64-88)	207 (100%, 98-100)
Facility has physicians charged full-time with management of TB patients	--	52 (25%, 20-31)
Facility has physicians charged part-time with management of TB patients	--	148 (71%, 65-77)
Patients with persistent cough in past two weeks	5.09 (2.41-7.77)	10.05 (8.12-11.97)
Number of TB patients being managed at end of calendar year 2014	1.05 (0.53-1.56)	9.43 (8.02-10.84)
Number of suspected TB patients in calendar year 2014	1.18 (0.47-1.9)	8.88 (7.23-10.52)
To where are suspected TB patients generally referred?		
Township Health Center	3 (7%, 2-18)	0 (0%)
County Hospital	3 (7%, 2-18)	50 (24%, 19-30)
City Hospital	0 (0%)	5 (2%, 1-6)
CDC	34 (77%, 63-87)	142 (69%, 62-75)
Rewards for discovered TB cases		
Facility receives reward	9 (20%, 11-33)	44 (21%, 16-27)
If yes, facility reward amount (*yuan*)	19.33 (9.33-29.33)	35.31 (26.02-44.6)
Physicians receive reward	9 (20%, 11-33)	73 (35%, 29-42)
If yes, physician reward amount (yuan)	19.33 (9.33-29.33)	18.67 (16.33-21.01)
Training		
Physicians have received Tuberculosis-specific training in 2014	30 (65%, 51-77)	164 (79%, 73-84)
If yes, times	2.83 (1.54-4.13)	2.49 (2.14-2.85)
Panel B: Doctor Characteristics		
	Village Physicians (N=46)	Township Physicians (N=207)
Practicing Physician Certificate	0 (0%)	127 (61%, 55-68)
Assistant Practicing Physician Certificate	6 (13%, 6-26)	48 (23%, 18-29)
Rural Physician Certificate	39 (85%, 72-92)	19 (9%, 6-14)
Age (years)	51.76 (49.13-54.39)	44.38 (42.94-45.81)
Male	40 (87%, 74-94)	178 (86%, 81-90)
Education, upper secondary or higher	4 (9%, 3-20)	124 (60%, 53-66)
Monthly salary (1,000 yuan)	2.16 (1.71-2.6)	3.42 (3.26-3.57)
Received Tuberculosis-specific training in 2014	30 (65%, 51-77)	58 (28%, 22-34)
If yes, times	2.83 (1.54-4.13)	1.57 (1.26-1.87)

Notes: Data are mean (95% CI) or n (%, 95% CI). Values are unconditional unless otherwise stated.
