# Supplementary material for: Tuberculosis detection and the challenges of integrated care in rural China: A cross-sectional standardized patient study
Source: PLoS Med. 2017 Oct 17;14(10):e1002405. doi: 10.1371/journal.pmed.1002405 (PMC5644979; doi:10.1371/journal.pmed.1002405)
Supplement: S2 Table — (PDF) [file pmed.1002405.s003.pdf]

S2 Table. Completion of Checklist Items

	Village Clinics	Township Health Centers	County Hospitals	Total
**Questions**				
Cough duration[1,2]*	0.61 (0.07)	0.85 (0.03)	0.76 (0.10)	0.80 (0.02)
(Produced) Sputum[1,2]*	0.43 (0.07)	0.58 (0.03)	0.38 (0.11)	0.54 (0.03)
Past tuberculosis[2]*	0.00 (0.00)	0.06 (0.02)	0.05 (0.05)	0.05 (0.01)
Family tuberculosis[2]*	0.00 (0.00)	0.03 (0.01)	0.05 (0.05)	0.03 (0.01)
Blood in sputum [1,2]	0.11 (0.05)	0.12 (0.02)	0.05 (0.05)	0.11 (0.02)
Fever (duration)[1,2]	0.26 (0.07)	0.40 (0.03)	0.48 (0.11)	0.38 (0.03)
Fever type[1,2]	0.24 (0.06)	0.24 (0.03)	0.24 (0.10)	0.24 (0.03)
Chest pain [2]	0.09 (0.04)	0.11 (0.02)	0.10 (0.07)	0.10 (0.02)
(Loss of) Appetite[1,2]	0.07 (0.04)	0.10 (0.02)	0.00 (0.00)	0.09 (0.02)
Lost weight[1,2]	0.00 (0.00)	0.02 (0.01)	0.05 (0.05)	0.02 (0.01)
Breathing difficulty[1,2]	0.00 (0.00)	0.14 (0.02)	0.05 (0.05)	0.11 (0.02)
Wheezing[1,2]	0.02 (0.02)	0.08 (0.02)	0.00 (0.00)	0.06 (0.01)
(Take) Any medicines[2]	0.46 (0.07)	0.63 (0.03)	0.29 (0.10)	0.57 (0.03)
Smoking[2]	0.07 (0.04)	0.07 (0.02)	0.05 (0.05)	0.07 (0.02)
Diabetes[2]	0.00 (0.00)	0.01 (0.01)	0.00 (0.00)	0.01 (0.01)
High blood pressure or hypertension[2]	0.11 (0.05)	0.04 (0.01)	0.00 (0.00)	0.05 (0.01)
HIV/AIDS[2]	0.00 (0.00)	0.00 (0.00)	0.00 (0.00)	0.00 (0.00)
Alcohol[2]	0.00 (0.00)	0.05 (0.02)	0.00 (0.00)	0.04 (0.01)
Age[2]	0.33 (0.07)	0.60 (0.03)	0.43 (0.11)	0.54 (0.03)
Family symptoms[2]	0.02 (0.02)	0.03 (0.01)	0.00 (0.00)	0.03 (0.01)
Have cough throughout the day[2]	0.17 (0.06)	0.14 (0.02)	0.05 (0.05)	0.14 (0.02)
Weakness[1]	0.00 (0.00)	0.09 (0.02)	0.05 (0.05)	0.07 (0.02)
Are there any night sweats present?[1]	0.13 (0.05)	0.14 (0.02)	0.14 (0.08)	0.14 (0.02)
**Exams**				
Weight[1,2]	0.00 (0.00)	0.00 (0.00)	0.00 (0.00)	0.00 (0.00)
Pulse[2]	0.20 (0.06)	0.08 (0.02)	0.00 (0.00)	0.09 (0.02)
Blood pressure[2]	0.09 (0.04)	0.08 (0.02)	0.00 (0.00)	0.07 (0.02)
Temperature[1,2]	0.59 (0.07)	0.62 (0.03)	0.19 (0.09)	0.58 (0.03)
Auscultation[1,2]	0.48 (0.07)	0.43 (0.03)	0.43 (0.11)	0.44 (0.03)
Chest radiograph[1,2]	0.17 (0.06)	0.35 (0.03)	0.90 (0.07)	0.36 (0.03)
Sputum smear test (Sputum AFB)[1,2]	0.00 (0.00)	0.04 (0.01)	0.05 (0.05)	0.04 (0.01)
HIV test[2]	0.00 (0.00)	0.00 (0.00)	0.00 (0.00)	0.00 (0.00)
Diabetes test[2]	0.00 (0.00)	0.00 (0.00)	0.00 (0.00)	0.00 (0.00)
Mantoux Tuberculin Skin Test (TST)[1,2]	0.00 (0.00)	0.00 (0.00)	0.00 (0.00)	0.00 (0.00)
Sputum culture test[1]	0.00 (0.00)	0.00 (0.00)	0.00 (0.00)	0.00 (0.00)

Notes: Data are proportion (se). [1] China Standard, [2] International (WHO) Standard, * essential items.
