# Supplementary material for: Tuberculosis detection and the challenges of integrated care in rural China: A cross-sectional standardized patient study
Source: PLoS Med. 2017 Oct 17;14(10):e1002405. doi: 10.1371/journal.pmed.1002405 (PMC5644979; doi:10.1371/journal.pmed.1002405)
Supplement: S3 Table — (PDF) [file pmed.1002405.s004.pdf]

S3 Table. Correlates of Correct Management, CXR, Referral, and Antibiotic Prescription of Standardized Patients among Village and Township Providers

	Correct Case Management	Chest X-Ray	Referral	Antibiotics
	(1)	(2)	(3)	(4)
Practicing Physician Certificate	2.76**	2.95**	2.33*	0.37**
	(0.01)	(0.01)	(0.08)	(0.02)
Facility has both X-ray equipment and staff able to operate	5.25***	3.83***	1.85	0.44**
	(0.00)	(0.01)	(0.27)	(0.05)
Receive reward for discovered TB patients	2.33**	2.17**	0.88	0.75
	(0.03)	(0.05)	(0.79)	(0.45)
Provider age (years)	0.94***	0.93***	0.92***	1.05**
	(0.00)	(0.00)	(0.00)	(0.03)
Male provider	0.48	0.40*	0.79	2.05
	(0.15)	(0.08)	(0.70)	(0.16)
Provider education, upper secondary or higher	0.44*	0.40*	0.49	1.44
	(0.05)	(0.05)	(0.15)	(0.40)
Provider month salary (1,000 yuan)	1.03	1.28	0.84	0.95
	(0.85)	(0.15)	(0.32)	(0.74)
Patient load (patients)	1.08	1.06	1.01	0.93
	(0.37)	(0.51)	(0.93)	(0.40)
Received TB training	0.96	0.97	1.34	0.78
	(0.92)	(0.93)	(0.53)	(0.52)
Village clinics	3.73**	1.92	2.33*	0.37**
	(0.05)	(0.32)	(0.08)	(0.02)
Number of Observations	253	253	216	253
Mean of Outcome	0.36	0.32	0.20	0.64

Notes: Results are reported as adjusted odds ratios. P-values are reported in parentheses under odds ratios. Correct case management is defined as a chest x-ray [CXR] or sputum test or referral. Regressions control for SP and county fixed effects. * p<0.1, ** p<0.05, *** p<0.01.
