# Supplementary material for: Tuberculosis detection and the challenges of integrated care in rural China: A cross-sectional standardized patient study
Source: PLoS Med. 2017 Oct 17;14(10):e1002405. doi: 10.1371/journal.pmed.1002405 (PMC5644979; doi:10.1371/journal.pmed.1002405)
Supplement: S4 Table — (PDF) [file pmed.1002405.s005.pdf]

S4 Table. Additional Statistics Comparing Vignettes and SP Visits.

	Desired (+) Unnecessary (–)	Perform-ance in Vignettes (N=243)	Perform-ance in SPs (N=243)	Correla-tion between Vignette and SP	Difference between Vignettes and SP			Odds Ratio		
**Consultation and Treatment**						95% CI	P-value		95% CI	P-value
**Percentage Mentioning TB**	+	47%	12%	0.23	-34%	(-0.42–-0.27)	[<0.001]	0.14	(0.08–0.22)	[<0.001]
**Correct management**	+	81%	36%	0.16	-45%	(-0.53–-0.38)	[<0.001]	0.10	(0.07–0.16)	[<0.001]
**Mean # Medicines Given or Prescribed**		2.22	2.45	0.01	0.23	(-0.17–0.62)	[0.257]	--	--	--
**Percentage Giving Any Antibiotic**	–	42%	66%	0.03	24%	(0.15–0.32)	[<0.001]	2.81	(1.92–4.1)	[<0.001]
**Percentage Referring Case**	+	48%	19%	0.19	-29%	(-0.37–-0.21)	[<0.001]	0.22	(0.14–0.34)	[<0.001]
**Examinations and Laboratory Evaluations (Percentage Completing)**										
**E - Chest X-Ray**	+	77%	32%	0.18	-45%	(-0.53–-0.38)	[<0.001]	0.11	(0.07–0.17)	[<0.001]
**E - Sputum AFB Test**	+	26%	4%	0.18	-22%	(-0.28–-0.16)	[<0.001]	0.10	(0.05–0.2)	[<0.001]
**E - X-ray and Sputum Test**	+	25%	4%	0.19	-21%	(-0.27–-0.15)	[<0.001]	0.10	(0.05–0.22)	[<0.001]
**E - X-ray or Sputum Test**	+	79%	32%	0.19	-47%	(-0.54–-0.39)	[<0.001]	0.10	(0.06–0.16)	[<0.001]
**E - Auscultation**	+	28%	44%	0.12	16%	(0.07–0.24)	[<0.001]	2.16	(1.45–3.23)	[<0.001]
**E - Temperature**	+	48%	61%	0.07	12%	(0.04–0.21)	[0.005]	1.72	(1.18–2.5)	[0.005]
**E - Weight**	+	0%	0%		0%	(-0.01–0)	[0.318]	1.00	--	--
**E - HIV Test**	+	1%	0%	-0.01	0%	(-0.02–0.01)	[0.565]	0.49	(0.04–5.61)	[0.563]
**E - Diabetes Test**	+	3%	0%	-0.01	-2%	(-0.04–0)	[0.057]	0.15	(0.02–1.32)	[0.087]
**E - Mantoux Test**	–	17%	0%		-17%	(-0.21–-0.12)	[<0.001]	1.00	--	--
**History Questions (Percentage Completing)**										
**Q - Cough Duration**	+	74%	80%	-0.01	6%	(-0.01–0.14)	[0.106]	1.44	(0.93–2.23)	[0.099]
**Q - Producing Sputum**	+	61%	55%	0.25	-6%	(-0.15–0.02)	[0.161]	0.76	(0.52–1.11)	[0.152]
**Q - Past TB**	+	5%	4%	0.32	-1%	(-0.05–0.02)	[0.513]	0.74	(0.31–1.79)	[0.504]
**Q - Family TB**	+	14%	2%	0.14	-12%	(-0.16–-0.07)	[<0.001]	0.09	(0.03–0.27)	[<0.001]
**Q - Blood in Sputum**	+	41%	11%	0.20	-30%	(-0.37–-0.23)	[<0.001]	0.16	(0.1–0.26)	[<0.001]
**Q - Fever Duration**	+	43%	37%	0.16	-7%	(-0.15–0.02)	[0.134]	0.74	(0.51–1.09)	[0.125]
**Q - Loss of Appetite**	+	19%	10%	0.21	-9%	(-0.15–-0.03)	[0.003]	0.43	(0.25–0.76)	[<0.003]
**Q - Weight Loss**	+	10%	2%	0.18	-8%	(-0.12–-0.04)	[<0.001]	0.15	(0.05–0.45)	[0.001]
**Q - Taken Any Medicine**	+	38%	60%	0.21	22%	(0.13–0.3)	[<0.001]	2.60	(1.78–3.8)	[<0.001]

Notes: For all items, the prefix "E" indicates examinations and laboratory evaluations; the prefix "Q" indicates history questions. Statistics reported in the "Correlation between Vignette and SP" column are Pearson coefficients for continuous variables and phi coefficients for binary variables. The gap calculation in the "Difference Column" is the result of a t-test comparing the average vignette performance with the average SP performance. P-values in brackets.
