# Supplementary material for: Tuberculosis detection and the challenges of integrated care in rural China: A cross-sectional standardized patient study
Source: PLoS Med. 2017 Oct 17;14(10):e1002405. doi: 10.1371/journal.pmed.1002405 (PMC5644979; doi:10.1371/journal.pmed.1002405)
Supplement: S5 Table — (PDF) [file pmed.1002405.s006.pdf]

S5 Table. Patient Selection into Health System Tiers with Symptoms of TB

	N	%
Panel A: Hypothetical		
If you or someone in your family had a cough and fever lasting for two weeks, would you go to see a doctor?	1963	97.09
If yes, which level of provider would you visit?		
Village clinics	897	45.7
Township Health Centers	606	30.87
County Hospitals	392	19.97
City Hospitals or higher	68	3.46
Panel B: Retrospective		
Did you or someone in your family have a cough and fever lasting for two weeks or more in the year 2015?	315	15.58
If yes, did you see a doctor?	294	93.33
If yes, which level of provider did you visit?		
Village clinics	130	44.22
Township Health Centers	83	28.23
County Hospitals	61	20.75
City Hospitals or higher	20	6.8

Source: Authors Data.

Notes: Nationally representative sample of 2,022 rural households.
