# Supplementary material for: Tuberculosis detection and the challenges of integrated care in rural China: A cross-sectional standardized patient study
Source: PLoS Med. 2017 Oct 17;14(10):e1002405. doi: 10.1371/journal.pmed.1002405 (PMC5644979; doi:10.1371/journal.pmed.1002405)
Supplement: S6 Table — (PDF) [file pmed.1002405.s007.pdf]

S6 Table. Simulation of System-level Management Outcomes with and without Managed Referrals (All Health Systems)

	**Patients Select Initial Provider Level***	**Managed Referrals§**	
		Start from VC	Start from THC
% Correctly Managed with Straight Referrals	35.9% (32.7-39.1)	8.8% (6.6-10.9)	34.8% (28.3-41.7)
% Correctly Managed with True Referrals	40.6% (37.8-43.4)	17.7% (15.2-20.2)	36.7% (30.1-43.7)

Notes: Data are % (95% CI). *Patient sorting in 'Patients Select Initial Provider Level' column based on a nationally representative sample of rural households: 45.7% at village, 30.87% at township, 23.43% at county. §Managed referrals refer to patients being required to initially visit providers at the village or township level. Simulations use observed referral rates for subsample of 46 "complete" health systems that include data on SP interactions at the village clinic level. For remaining 163 health systems, village clinic referral rates are imputed with the average referral rates of observed village clinics.
