# Supplementary material for: Tuberculosis detection and the challenges of integrated care in rural China: A cross-sectional standardized patient study
Source: PLoS Med. 2017 Oct 17;14(10):e1002405. doi: 10.1371/journal.pmed.1002405 (PMC5644979; doi:10.1371/journal.pmed.1002405)
Supplement: S7 Table — (PDF) [file pmed.1002405.s008.pdf]

S7 Table. Simulation of Out-of-Pocket Costs with and without Managed Referrals

	**Patients Select Initial Provider Level***	**Managed Referrals§**	
		Start from VC	Start from THC
% Correctly Managed with Straight Referrals †	61.24 (49.76-72.71)	33.96 (25.76-42.15)	60.58 (40.42-80.73)
% Correctly Managed with True Referrals	65.37 (50.69-80.05)	43.00 (24.83-61.17)	60.58 (40.42-80.73)

Notes: Data are mean (95% CI). 6.5 RMB = $1. *Patient sorting in 'Patients Select Initial Provider Level' column based on a nationally representative sample of rural households: 45.7% at village, 30.87% at township, 23.43% at county. §Managed referrals refer to patients being required to initially visit providers at the village or township level. † "Straight Referrals" refers to referrals only being allowed to the next highest tier. "True Referrals" allow for bypassing.
