# Supplementary material for: Tuberculosis detection and the challenges of integrated care in rural China: A cross-sectional standardized patient study
Source: PLoS Med. 2017 Oct 17;14(10):e1002405. doi: 10.1371/journal.pmed.1002405 (PMC5644979; doi:10.1371/journal.pmed.1002405)
Supplement: S1 Fig — (PDF) [file pmed.1002405.s009.pdf]

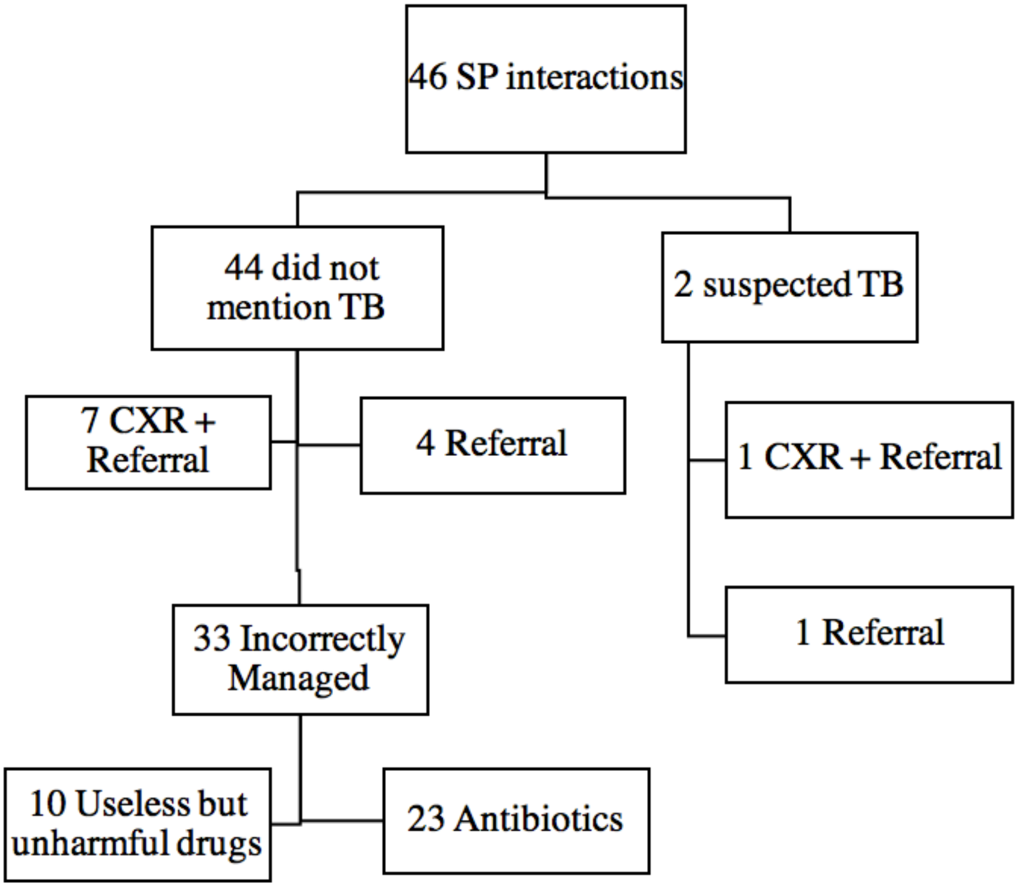

S1a Fig. Case Management of SPs in Village Clinics

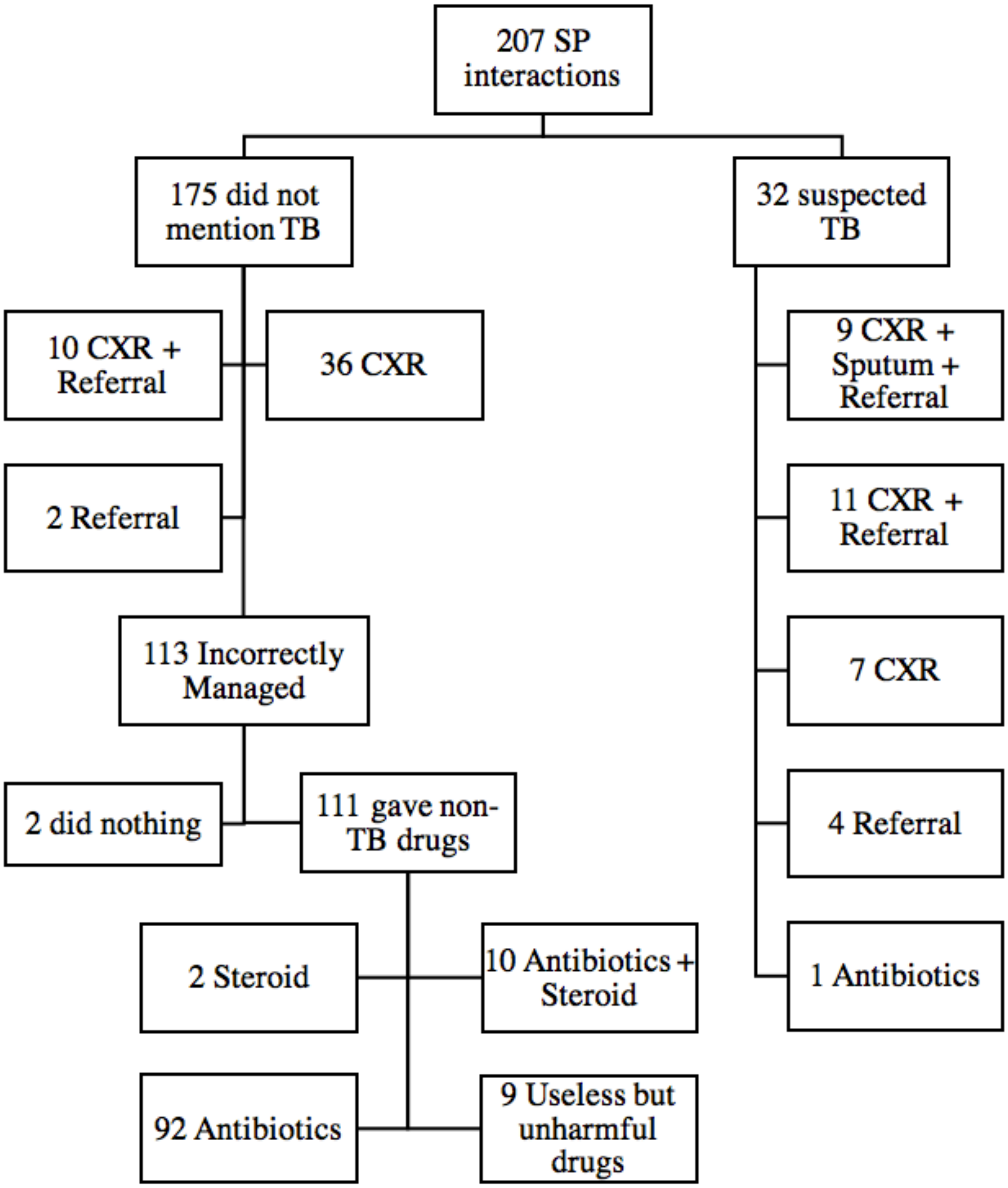

S1b Fig. Case Management of SPs in Township Health Centers

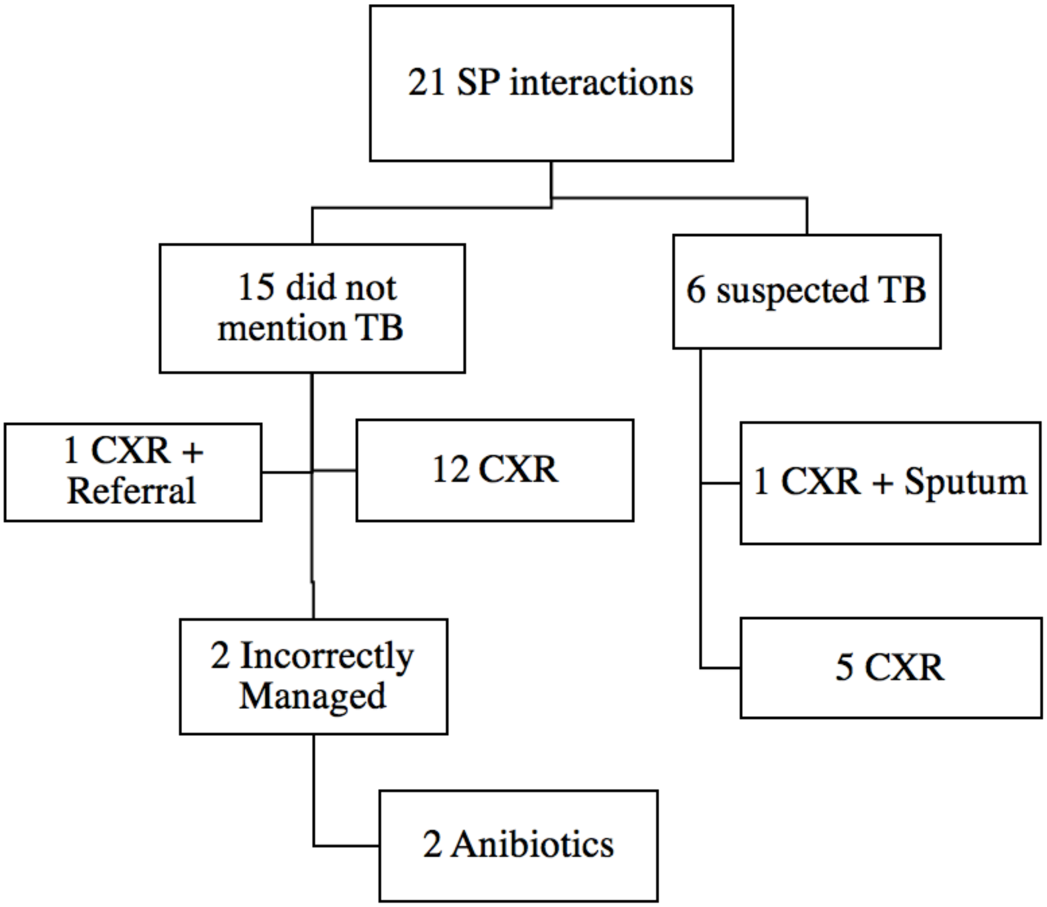

S1c Fig. Case Management of SPs in County Hospitals
