# Supplementary material for: Tuberculosis detection and the challenges of integrated care in rural China: A cross-sectional standardized patient study
Source: PLoS Med. 2017 Oct 17;14(10):e1002405. doi: 10.1371/journal.pmed.1002405 (PMC5644979; doi:10.1371/journal.pmed.1002405)
Supplement: S2 Fig — (PDF) [file pmed.1002405.s010.pdf]

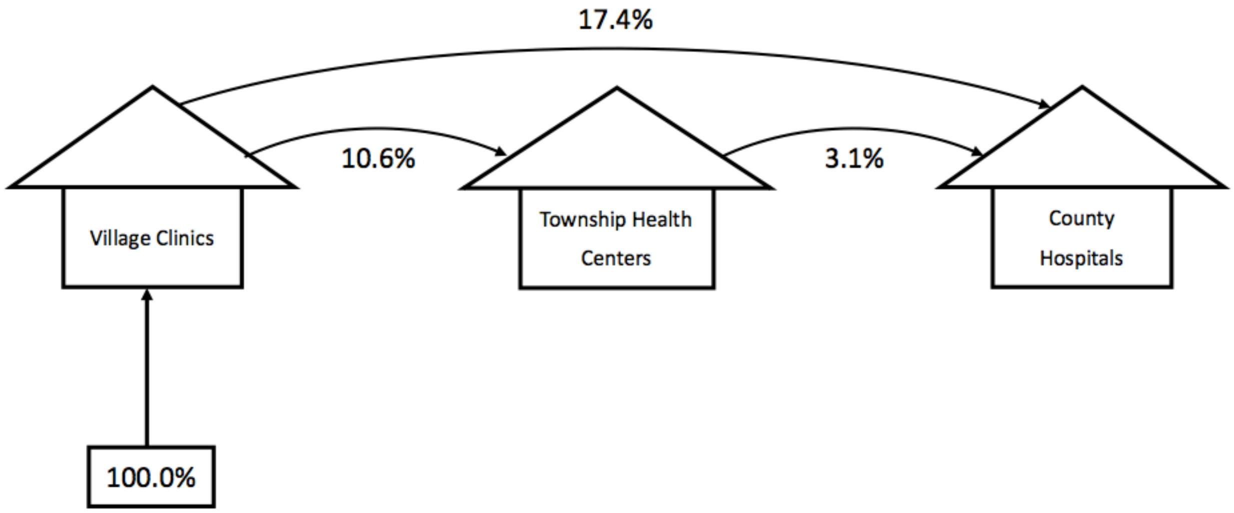

S2a Fig. Estimated Patient Pathways under Gatekeeping from the Village Level

Notes: For each referral pathway, figure shows percentage of total patient population following each path calculated using SP results.

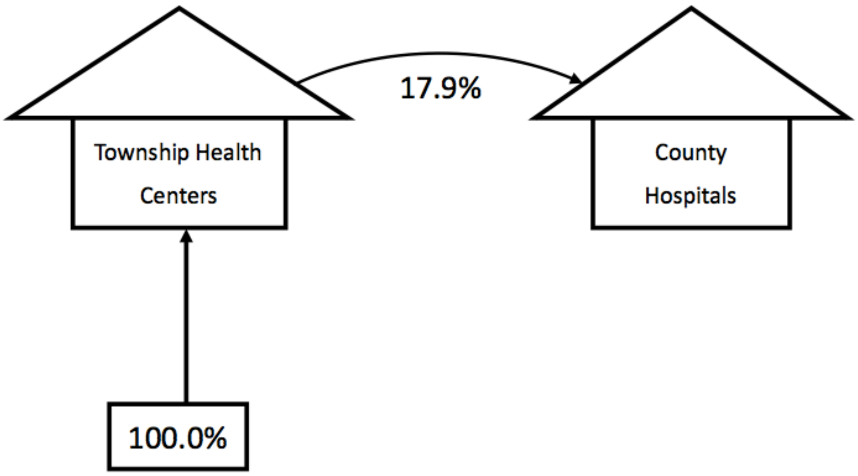

S2b Fig. Estimated Patient Pathways under Gatekeeping from the Township Level

Notes: For each referral pathway, figure shows percentage of total patient population following each path calculated using SP results.
