# Supplementary material for: Tuberculosis detection and the challenges of integrated care in rural China: A cross-sectional standardized patient study
Source: PLoS Med. 2017 Oct 17;14(10):e1002405. doi: 10.1371/journal.pmed.1002405 (PMC5644979; doi:10.1371/journal.pmed.1002405)
Supplement: S1 Text — (PDF) [file pmed.1002405.s011.pdf]

S1 Text. Methodological Appendix

Supplement to:

Tuberculosis Detection and the Challenges of Integrated Care in Rural China: A Cross-sectional Standardized Patient Study

Authors: Sean Sylvia, Hao Xue, Chengchao Zhou, Yaojiang Shi, Hongmei Yi, Huan Zhou, Scott Rozelle, Madhukar Pai, Jishnu Das

Corresponding author:

Chengchao Zhou, PhD

Institute of Social Medicine and Health Administration

School of Public Health

Shandong University

44 Wenhua West Road

Jinan City, Shandong Province 250012, China

Email: zhouchengchao@sdu.edu.cn

1. Provider Sample Selection

The sample of providers for this study was selected to be representative of health systems (triplets, or referral chains, of village, township, and county-level hospitals) in three city-level prefectures, one in each of Shaanxi, Sichuan, and Anhui provinces. We used the following procedure to sample health systems: First, across the three prefectures, we randomly sampled 21 of 24 rural counties and included the primary county-level hospital in each sampled county. Next, 10 THCs were randomly sampled within each county. Because even counties designated as `rural' have an urban township housing the county seat, we excluded the health center of the urban township. One county only had 9 rural townships, yielding a sample of 209 of the total 311 THCs in the 21 sample counties. Finally, we randomly selected one VC associated with each sampled THC for a total of 209 VCs. This village was selected from an administrative list of villages in each township. Because we anticipated that some villages would not have a village clinic, a backup village was randomly selected in each township. Out of the 209 originally-sampled villages, 22% had no village clinic and was replaced with the backup.

Standardized patients presenting the TB case were sent to all sampled county level providers, all sampled township level providers, and a randomly-chosen 49 of the 209 village clinics included in the sample. SPs presenting other cases (diarrhea and angina) were sent to remaining village clinics. Only one SP was sent to village clinics to minimize the risk that SPs were identified as fake patients.

2. Standardized Patients

2.1 Description of SP Case

The SP case was designed to depict a classic case or presumed TB. The SP begins the interaction with physicians using the opening statement: "Doctor, I have a cough that is not improving and a fever." The SP then answers any questions asked by the physician and receives any (non-invasive) exams. Upon appropriate questioning by the provider, the SP reveals symptoms

consistent with a classic case of presumed tuberculosis including a cough duration of 2-3 weeks, fever with night sweats and loss of appetite and weight.

2.2 SP script development

The SP case used in this study was adapted from an earlier validation study in India (Das et al. 2015). That study demonstrated (1) participation in the study had minimal to no risk for the SPs or health care providers, (2) the likelihood of SP detection among visited providers was low, confirming that SPs were considered real by health providers who were visited, and (3) the abilities of the SPs to recall what occurred during the interaction was strongly correlated with what actually happened. Additionally, because the SPs pay the fees requested by the healthcare provider, there is no loss to provider income from participation in the study.

For the purposes of the current study, the SP case was adapted to the Chinese context with the help of an advisory panel consisting of TB experts with the China CDC Institute for Tuberculosis Control and Prevention and physicians employed in the public health system. Adaptation of the scripts included: (1) ensuring that the clinical presentation of the SP would be interpreted clearly given local context; (2) ensuring that SP responses were prepared for any likely questioning by physicians (3) developing SP interaction protocols given clinical settings in China, including protocols for avoiding invasive procedures; (4) developing detailed background histories for SPs to minimize the threat of SP detection as fake patients. Additionally, because local dialects varied across the three regions of the study, scripts with alternative phrasing to match the local dialect (where appropriate) were developed from a version in standard Mandarin. These were small alterations to phrasing or vocabulary, and were chosen to convey the exact information as the standard script. SP script adaptations took place as an iterative process including field pretesting (in local but not project areas) with 6 pre-trained SPs.

The SP Script (Including background and dialog) is available for download at https://seansylvia.web.unc.edu/sp-case-script-tuberculosis/. See Das et al. (2015) for the original script used in the validation study.

2.3 SP recruitment and characteristics

To ensure low likelihood of detection among visited providers, SPs were recruited from local areas. This meant that SPs were similar to patients typically seen by the clinics in terms of language (dialect), mannerisms, and dress. A total of 21 individuals (10 males and 11` females) from an initial group of 24 were hired and trained as SPs from three provinces (7 from each province). The SPs, although recruited specifically to fit the TB case in terms of health and physical characteristics, differed in age, gender height and weight. Our recruitment standard for TB SPs was that they be around 35 years old, average weight and height, and healthy with no obvious signs of illness or other conditions that could prejudice diagnoses. The average age of recruited SPs was 37, the youngest was 28 and the oldest 43.

2.4 SP training

All SPs underwent a centralized intensive two-week training. The aim of SP training was to ensure that they (a) correctly presented the cases in a consistent way, (b) correctly recalled the interaction with physicians, (c) avoided detection and (d) SPs would be able to complete interactions safely without being exposed to invasive tests or procedures.

These aims were achieved through classroom training in case presentation and testing of recall, as well as mock interviews and dry runs that were supervised in the field. The training started with a focus on the cases and the development of scripts and proceeded to memorization and appropriate role-playing. SPs were taught to internalize completely the characters and the details of their background stories. Mock interviews were conducted with trainers as well as physicians working with the research team. These mock interviews initially asked only potentially clinically relevant questions, but in later rounds added additional questions about family or neighborhood to ensure that SPs could answer appropriately and convincingly. Mock interviews also simulated "threats" of invasive procedures.

In the final week of training, SPs conducted supervised dry runs in clinics nearby the training site. Since it is common in China that more than one patient is present in the examination room at the same time, dry runs were conducted in which a supervisor was present and thus could watch the interaction and offer corrections later. Dry runs were also conducted during a two-day practice round in local areas to ensure that the SPs were accustomed to local conditions before starting data collection.

Enumerators accompanying SPs also attended the full training in order to familiarize themselves with the survey process and the SP visit.

2.5 Assignment of SPs to providers

All SPs were randomly assigned to clinics. Each survey team (comprising of an TB SP and an enumerator) were randomly assigned to two counties within each province. Within each county, teams were assigned a random half of sample townships. SPs were never assigned to their home township where they would risk being recognized.

Each survey team first visited their assigned townships (both township health centers and village clinics in each township) in their first assigned county and then traveled to their second assigned county. County hospitals were visited by the second team assigned to each county.

Within each facility, SPs visited the doctor following the normal procedures for any walk-in patient. Given a choice of which doctor to visit, SPs randomly chose a doctor following a pre-determined randomization protocol. In county hospitals, where patients can choose doctors by specialty, SPs visited generalists. Our results therefore approach the care a walk-in patient would receive at each of the sampled facilities.

2.6 Consent

Informed consent was obtained verbally from all providers participating in the study. To prevent influence on the study, a procedure was approved whereby providers consented to SP visits "at

some point in the next six months." Consent from village and township providers was obtained as part of the facility survey approximately 5 weeks before SP visits using the script below. Consent for county providers was obtained through communications with providers. All individuals who participated as SPs were trained to protect themselves from any invasive tests or procedures.

Following the conclusion of the baseline survey, consent was obtained verbally from providers using the following script: *"At some point during the next six months, we may send a fake patient may visit your facility to seek care. If you believe that a patient is a fake patient, please record the patients name, symptoms, the timing of the visit, but do not directly ask patients if they are a fake patients. Do you agree for a fake patient to visit your facility?"*

2.7 Drug Identification

To get full information about the drugs and cost for each interaction, SPs purchased any medications prescribed and paid providers their usual fee. After each visit, enumerators packed all the drugs for each case in an individual bag and labeled all the information related to drugs in it.

In the case of drugs prescribed to be taken intravenously, the protocol was designed to allow SPs to avoid being administered the IV while also recording the drugs to be administered. If an IV was prescribed, SPs paid for the IV and took the written prescription but left before being administered the IV (indicating that they would soon return). If the written prescription was illegible, SPs asked pharmacy staff the contents of the IV. If there were no pharmacy staff, SPs asked physicians the contents directly. IVs were prescribed in 11% of village clinic interactions, 28% in township health centers, and 5% in county hospitals.

All labelled medicines prescribed by the pharmacies were digitized and stored and then coded by enumerators with the assistance of consulting doctors and pharmacologists. Blinded from any provider details, they identified and categorized medicines as steroids, anti-TB drugs,

fluoroquinolones, or other antibiotics. Loose or unlabeled pills were dispensed in 10% of THC interactions and 37% of village clinic interactions. Less than 5% of these drugs could not be identified (we did not perform chemical testing).

3. Detection of SPs

To assess the rate at which SPs were detected (as fake patients), a detection survey was launched 2-3 weeks following SP visits. Physicians were told at the time of giving consent to participate in the study to record information on any patient that suspected as a fake patient. During the detection survey following SP visits, physicians were asked whether they suspected anyone as a standardized patient and, if so, to report the characteristics of detected SPs and the specific symptoms provided by the suspected patient.

All sampled village and township physicians completed the detection survey. Of these 9 (4%) reported that they suspected someone as an SP. Of the 9 total SP detections, 6 (2%) physician descriptions matched the standardized patient. As anticipated, the SP detection rate in village clinics was higher than in township health centers (4% vs 2%). In no cases did the provider voice suspicion during the interaction.

4. Vignettes

Clinical vignettes were administered to village and township doctors in September 2015, 2-3 weeks after SP visits. Vignettes were administered by two enumerators: one playing the role of the patient and the other providing instructions and recording the interaction. At the start, providers were asked to proceed as they would with a real patient and told that the patient would answer any questions and comply with any instructions. In contrast to SP visits, physicians participating in vignettes know they are being tested in the case of vignettes and are thus likely to perform to the extent of their knowledge.

Vignettes were administered to all doctors in village clinics and township health centers who were visited by SPs. Vignettes given to each doctor matched the disease cases depicted by SPs

visiting that doctor. To match vignettes to the correct doctors, the identity of visited doctors was confirmed at the time of SP visits. SPs were trained to obtain the doctors name either from the doctors name card, office titles, prescriptions, pharmacy staff, showcase windows common in township hospitals with the names and photos of each doctor. SPs were also shown pictures of each doctor (taken during the facility survey) by enumerators upon exiting the facility. If SPs visited a doctor who was not present during facility survey (which happened rarely in practice), enumerators recorded the name, gender, approximate age and the characteristics of this doctor and coded as a new doctor. This doctor was then administered a basic survey at the time of the vignette survey.

5. Supplementary Household Survey Data on Patient Sorting

The supplementary household survey data on patient sorting was from a dataset collected by authors in April 2016. The survey was designed to be nationally representative of rural households. Sampling followed a multistage cluster sampling procedure, first randomly sampling five provinces (Jiangsu, Sichuan, Shaanxi, Jilin and Hebei), five counties within each province, two townships within each county, and two villages within each township. The final sample consists of 2024 rural households across 100 villages. Detailed information on the survey can be found in Zhang et al. (2016).

We asked each household head two sets of questions to assess how patients sort across health system tiers with symptoms of TB matching the SP case. The first set asked hypothetically: “If you or someone in your family had a cough and fever lasting for two weeks, would you go to see a doctor? If yes, what level of provider would you visit?” The second set of questions were analogous, but asked retrospectively about the household’s experience in 2015: “Did you or someone in your family have a cough and fever lasting for two weeks or more in the year 2015? If yes, did you see a doctor? If yes, what level of provider did you visit?” Responses to these questions are summarized in S4 Table.

6. Case management conditional on diagnoses

S2a Fig, S2b Fig and S2c Fig show case management outcomes at each provider level, conditional on diagnoses given by physicians. Separately for physicians who did and did not mention TB as a potential diagnosis, these figures show the number of SPs who were suggested a CXR, sputum tests, or were referred to upper level providers. We also show drug prescriptions focusing on (non-TB) antibiotics and steroids.

Correct diagnoses (mention of TB) were more common in higher level clinics (4% in village clinics, 15% in township health centers and 29% in county hospitals). For the correctly diagnosed cases, most were correctly managed. Among physicians not suspecting TB as a possible diagnosis, 25% were nevertheless correctly managed at the village level, 27% in township health centers, and 87% in county hospitals due to suggestion of CXR or referral. Among cases incorrectly managed, nearly all were prescribed drugs. At the village level, antibiotics were prescribed in 70% of these cases. In township health centers, antibiotics were prescribed in 92% of these interactions and steroids in 11%. Of the two interactions in county hospitals that were incorrectly managed, both were prescribed antibiotics.

7. Planned Analyses and Deviations

From conception, this study aimed to assess 1) provider's adherence to national and international standards of care (including specifically rates of correct case management and adherence to predetermined checklists), 2) how provider and facility characteristics related to correct management, components of correct management, and drug prescriptions, and 3) the "know-do gap" between provider practice and knowledge. The definitions of the primary outcomes of the study, including "correct management", as well as secondary outcomes and the standards used to assess the clinical process (checklists of suggested questions and exams) were determined prior to data collection. These definitions and the set of outcomes used closely follow the analysis in a previous validation study by the authors conducted in India (Das et al. 2015). The method of estimating the "know-do gap" (comparing vignettes and SP visit outcomes) was also determined prior to data collection and follows previous studies by the authors. The specific approach to comparing vignettes and SP visit outcomes (using OLS with

county fixed effects) was determined at the analysis stage; however, this was not based on results of any previous analysis.

The characteristics included in regressions testing the correlates of case management outcomes (shown in Fig 3 and S3 Table) were determined at the analysis phase and further augmented with the variable “Facility has both X-ray equipment and staff able to operate” in response to reviewer comments. While the variables included were determined at analysis, and should be considered exploratory, decisions to include or exclude variables from this analysis was based on theory and not influenced by results of different specifications.

The decision to conduct simulations of system-level management outcomes was also made after data on the primary outcomes was collected; however, this decision was not based on the results of any initial analysis.

References

Das J, Kwan A, Daniels B, et al. Use of standardised patients to assess quality of tuberculosis care: a pilot, cross-sectional study. *The Lancet Infectious Diseases*, 2015, 15(11): 1305–1313.

Zhang, L, Li, S, Yi, H, et al. Correlation Between New Cooperative Medical Scheme Policy Design and Catastrophic Medical Payment: Evidence From 25 Counties in Rural China. Asia Pacific Journal of Public Health, 2016, 28(1), 26–38.
